# Exploration of a novel and efficient source for production of bacterial nanocellulose, bioprocess optimization and characterization

**DOI:** 10.1038/s41598-022-22240-x

**Published:** 2022-11-02

**Authors:** Noura El-Ahmady El-Naggar, Sahar E. El-Malkey, M. A. Abu-Saied, A. B. Abeer Mohammed

**Affiliations:** 1 Department of Bioprocess Development, Genetic Engineering and Biotechnology Research Institute, City of Scientific Research and Technological Applications (SRTA-City), New Borg El- Arab City, Alexandria, 21934 Egypt; 2 Microbial Biotechnology Department, Genetic Engineering and Biotechnology, Research Institute, University of Sadat City, Sadat City, Egypt; 3 Polymeric Materials Research Department, Advanced Technology and New Materials Research Institute, City of Scientific Research and Technological Applications (SRTA-City), New Borg El-Arab City, Alexandria, 21934 Egypt

**Keywords:** Nanoparticles, Biotechnology, Biopolymers

## Abstract

The demand for bacterial nanocellulose is expected to rise in the coming years due to its wide usability in many applications. Hence, there is a continuing need to screen soil samples from various sources to isolate a strain with a high capacity for bacterial nanocellulose production. *Bacillus* sp. strain SEE-12, which was isolated from a soil sample collected from Barhiem, Menoufia governorate, Egypt, displayed high BNC production under submerged fermentation. *Bacillus* sp. strain SEE-12 was identified as *Bacillus tequilensis* strain SEE-12. In static cultures, BNC was obtained as a layer grown in the air liquid interface of the fermentation medium. The response surface methodology was used to optimise the process parameters. The highest BNC production (22.8 g/L) was obtained using 5 g/L peptone, 5 g/L yeast extract, 50%, v/v Cantaloupe juice, 5 g/L Na_2_HPO_4_, 1.5 g/L citric acid, pH 5, medium volume of 100 mL/250 mL conical flask, inoculum size 5%, v/v, temperature 37 °C and incubation time 6 days. The BNC was purified and characterized by scanning electron microscopy (SEM), Energy-dispersive X-ray (EDX) spectroscopy, differential scanning calorimetry (DSC), X-ray diffraction (XRD), Fourier transform infrared spectroscopy (FTIR), thermogravimetric analysis (TGA) and transmission electron microscopy (TEM).

## Introduction

The term “nanocellulose” refers to cellulose in the nanometer range. This could be cellulose cellulose nanofibers (CNF), bacterial nanocellulose (BNC) or nanocrystals (CNC), which is nano-structured cellulose synthesized by bacteria^1,2^. The BNC is a natural nanoscale biodegradable linear biopolymer mainly synthesized by some species of bacteria through microbial fermentation. The bacterial cellulose is comprised of glucose monomers that are linked together by β-1,4-glycosidic linkages and has the chemical formula (C_6_H_10_O_5_)n). Bacterial cellulose (BC) presents the same chemical formula as plant cellulose but exhibits superior physical and chemical properties over plant cellulose^3^. The BNC is secreted in the form of pellicles or sheets comprised of ultrafine ribbons of 4–5 nm in thickness of cellulose filaments and 30–50 nm in width^4^. The BNC displays unique properties, that include excellent biocompatibility and biodegradability, lack of toxicity, high tensile strength, high flexibility, chemical and heat shock resistance, ease of sterilization, fibrous network structure, higher water holding capacity, high crystallinity, extreme purity (free of lignin, hemicellulose, and pectin present in plant cellulose), and a high degree of polymerization and renewable properties^4^. The production of bacterial cellulose has recently attracted significant attention due to its numerous applications in a variety of fields, including medicine, pharmaceuticals, food industries, pulp and paper industry, textile industry, mining, waste treatment, etc. Among the most well-known industrial applications of bacterial cellulose include diaphragms made of dried cellulose sheets for electro-acoustic transducers^5^, eco-friendly sustainable fabric with new sensibilities in the fashion fabric industry^6^.

BC-based nanocomposites are obtained by combining a variety of components (such as biopolymers and nanoparticles). BC-nanocomposites have been used in several biomedical applications, including wound dressing material (artificial skin) for people who have been severely burned^7^, fabrication of artificial blood vessels for use in microsurgery^8^, cartilage and bone^9^, in vitro and in vivo drug delivery systems, scaffolds for tissue engineering, and vascular grafts^10^ cardiovascular system, antimicrobial and antiviral films^11^.

However, these applications of BNC are limited due to the relatively expensive cost. The high price is due to the high cost of the culture medium, as well as the poor production rate of well-known bacterial strains on an industrial scale^10^. One way to reduce the total cost of producing BNC is to identify a low-cost and renewable carbon source through bioprocess optimization of the growth medium that can increase the BNC yield^12^. In recent years, several experimental studies have been conducted to reduce the production costs by using efficient and cost-effective culture medium and various waste products such as rotten fruits^13^, fruit juices as citrus juice^14^ kiwifruit peel hydrolysate (Güzel and Akpınar, 2020)^15^, apple peel hydrolysate^15^, pineapple peel waste^16^, recycled paper sludge hydrolysate^17^ and an extract of banana peel waste^16^. Food wastes contain mostly carbon sources in the form of simple sugar or complex fiber. Take pineapple peel juice derived from pineapple waste as an example; it has a high sugar content (73.76 g/L of total sugar), which is primarily made up of glucose, sucrose, and fructose. Conversely, the peels of pineapple are cellulose fibers that can be further processed to produce simple sugar^17,18^.

Identification of a cost-effective culture medium for BNC production is one of the most crucial and challenging aspects of the fermentation process. Submerged fermentation is an advantageous technique for BNC production that provides a low-cost end product to promote industrialization and commercialization of the BNC^19^. It is important to optimize the fermentation process in order to increase BNC production while keeping production costs low. Traditionally, fermentation processes have been improved in order to achieve maximum yields by changing one independent variable at a time while keeping the others constant. Traditional optimization (one-at-a-time method) is complicated and time-consuming, particularly when multiple variables are being screened and does not consider the complex interactions that exist between the various variables^20^. As a result, the statistical optimization involves assessment of many variables simultaneously in a restricted number of experimental runs, and it has the advantage of quantifying possible interactions among different variables. It is a less time consuming process and minimises inaccurate interpretation that occurs in one factor at a time optimization^21^. Both one-factor-at-a-time approach and then response surface method were applied to optimize BC production from *Gluconacetobacter xylinus* TJU-D2^22^ and *Komactobacter intermedius*^23^.

*Gluconacetobacter xylinus* (previously named *Acetobacter xylinum* and *Acetobacter xylinus*) has been reported to have the highest capacity for extracellular bacterial cellulose production^24^. *Gluconacetobacter xylinus* is an acetic acid bacteria belonging to the family *Acetobacteraceae*. It is rod‐shaped, obligately aerobic and Gram‐negative^25^. The prominent characteristic of this bacterium is its potential to synthesize cellulose, with a high crystallinity degree that distinguishes it from plant cellulose^26^. As a result, *Gluconacetobacter xylinus* has been used as a model organism for extracellular bacterial cellulose production in numerous studies^26–28^.

Several species of Gram‐negative bacteria have been reported to have the capacity for bacterial cellulose production^16,17,26,30–37^. In contrast, the production of BC by Gram-positive bacteria has only been reported from a limited number of species including: *Bacillus licheniformis* strain ZBT2^38^, *B. amyloliquefaciens* ZF-7^39^, *Bacillus velezensis* strain SMR^40^, *Lactobacillus hilgardii* IITRKH159^29^, *Leifsonia* sp.^41^, *Leifsonia* sp. CBNU-EW3^42^ and *Rhodococcus* sp. MI 2^43^. Numerous efforts have been made to isolate highly productive isolates for the bacterial cellulose production for industrial use^44^.

The goal of this study was to isolate bacteria with a high capacity for cellulose production; the isolated strain was characterized by biochemical and morphological tests in addition to 16S rRNA sequencing; and to evaluate effects of ten different carbon sources on the BNC production by *Bacillus tequilensis* strain SEE-12. Statistical approaches have been used to improve and maximize BNC production for commercialization and industrial applications. The purity, structure, and characteristics of the BNC, including surface morphology and nanocellulose diameter by scanning electron microscopy analysis (SEM) and degree of polymerization by X-ray diffraction (XRD) and thermal decomposition behaviour by Thermogravimetric analysis (TGA) and differential scanning calorimetry (DSC) were investigated.

## Materials and methods

### Microorganisms and growth conditions

Bacterial isolate SEE-12 was isolated from a soil sample collected from Barhiem, Menoufia governorate, Egypt using M9 minimal salt medium supplemented with Locust bean gum (LBG) and yeast extract of the following composition (g/L): 0.5 of Locust bean gum (LBG); 6.78 Na_2_HPO_4_; 3 KH_2_PO_4_; 1 NH_4_Cl; 0.5 yeast extract; 0.5 NaCl; 0.1 mL of 1 M CaCl_2_; 2 mL of 1 M MgSO_4_; 20 Agar and distilled water up to 1L^45,46^. Nystatin was added to the medium as a broad-spectrum antifungal agent to prevent the growth of fungi. Petri plates of the previous medium were inoculated with a loopful of soil suspension, then incubated for 24 h at 30 °C. The inoculated plates were examined for the appearance of bacterial colonies. The bacterial colonies that exhibited culture features typical of *Bacillus* species, such as thick and opaque; cream-colored, round or irregular were subcultured and purified on nutrient agar plates. The purified *Bacillus* species were then screened for their ability for nanocellulose production before being identified.

### Inoculum preparation

For preparing the inocula, the bacterial cells were grown in 250 mL Erlenmeyer conical flasks containing 100 mL of the synthetic medium composed of (g/L): glucose 20, yeast extract 5, peptone 5, Na_2_HPO_4_ 2.7 and citric acid 1.15, pH was adjusted to be 5^47,48^. The medium was autoclaved for 20 min at 121 °C. The bacterial cells were grown, after inoculation, under static conditions for 24 h at 30 °C.

### Screening the potential of different bacterial isolates for the production of the BNC

The potential of different bacterial isolates for BNC production was screened in static-flask cultures using two fermentation culture media; the first (Medium 1) was a growth medium containing (g/L): yeast extract 5, peptone 5, glucose 20, citric acid 1.15, Na_2_HPO_4_ 2.7 and distilled water up to 1L; the initial pH was adjusted to 5 using NaOH 1 M^47,48^ and the second (Medium 2) was modified standard Hestrin-Schramm medium^47^ containing (g/L): yeast extract 5, peptone 5, glucose 20, Na_2_HPO_4_ 2.67 and citric acid 1.5 or until the initial pH was 3.6^37^. A 10% (v/v) stock culture was used as an inoculum to inoculate the broth medium and grown at 30 °C under static conditions for 14 days. The bacterial isolates that were able to produce pellicle at the air–liquid interface of the culture medium were considered BNC-producing isolates. The bacterial isolate chosen to produce BC in the subsequent step based on the maximum thickness of the pellicle and the highest BC yield expressed in g/L. For further studies, BNC-producing isolates were preserved in vials containing a 20% glycerol solution and kept at − 20 °C.

### Harvesting, purification and quantification of the BNC

After fermentation, the BNC layers which were synthesized and secreted in contact with the air as the exopolysaccharides were harvested after a period of seven to fourteen days of cultivation and treated by the protocols of Masaoka et al.^49^ and Wu et al.^50^ (Supplementary Material). The production yield (in g/L dry mass) was determined.

### Identification of the bacterial isolate

The most promising bacterial isolate (strain SSE-12) was identified according to its morphological, Gram staining, spore formation, and biochemical tests. The bacterial isolate (strain SSE-12) was molecularly identified using 16S rRNA sequencing using the protocol of El-Naggar et al.^51^ (Supplementary Material). MEGA version X software was used to construct the phylogenetic tree using the neighbour-joining method^52^.

### Production of the BNC on different carbon sources

The influence of various carbon sources on the production of BNC by a selected strain was evaluated using the two culture media mentioned above. The fermentation was performed in liquid culture media under static conditions using 250 mL Erlenmeyer flasks, each containing 100 mL of seed culture medium. Ten carbon sources (glucose, glycine, mannitol, fructose, starch, ribose, xylose, sucrose (with a concentration of 2%), Cantaloupe juice, and *Ulva lactuca* biomass extract “(%, v/v)”, were sterilized and added to the sterilized medium to determine a more appropriate source of carbon to produce the BNC for up to 14 days. The amount of the BNC produced (in g/L dry mass) was determined.

The Cantaloupe fruits have been processed into clarified juice by squeezing the frozen and thawed Cantaloupe flesh in a blender with the peel removed and then filtered. *Ulva lactuca* biomass was collected and extensively rinsed with seawater to eliminate any contaminants, adherent sand particles, or epiphytes. Under ambient temperature, *Ulva lactuca* biomass was thoroughly washed under running tap water to eliminate salts and then dried to remove moisture. The extraction method according to the modified procedures described by Latique et al.^53^. 20 g of the dried crushed algal biomass was mixed with 100 mL of distilled water in a 250 mL flask, and boiled separately for one hour in a water bath, then the mixture was filtered to remove debris. This filtrate represented a 100% algal crude extract.

### Selection of significant variables using plackett–burman design (PBD)

PBD^54^ is a two-factorial design that defines different physico-chemical factors necessary to produce high levels of the response with respect to their main effects^55^. In the present work, ten variables were chosen to be screened by PBD in addition to one dummy variable (Supplementary Material). The Plackett–Burman design does not define the mutual interactions between the process variables; rather, it is employed to screen for and identify significant variables that influence the response^56^. So, the face-centered central composite design (FCCCD) was used to define the levels of the most significant variables variables and to investigate the interaction effects among multiple significant variables.

### Face centered central composite design (FCCCD)

Face-centered central composite design (FCCCD) is an efficient design that is widely used in optimization processes because it provides a sufficient amount of information for validating the accuracy of the model without requiring a large number of experimental runs, thereby lowering the overall cost of the experiment^57^. Based on the results of the Plackett–Burman experiment, FCCCD was used to find the best levels and to study the interaction effects among the most significant independent variables that affect BNC production. These variables, namely pH (X_1_), Cantaloupe juice (X_2_) and incubation time (X_3_). A total of 20 runs were performed in order to optimize the levels and to study the interaction effects among the chosen factors on the BNC synthesis by *Bacillus tequilensis* strain SEE-12 (Supplementary Material).

### Solubility in water and standard organic solvents

The solubility of BNC produced by *Bacillus tequilensis* strain SEE-12 was investigated in water, standard organic solvents (water, ethanol, chloroform, DMSO, propanol, xylene, methanol, butanol, isopropanol, acetic acid), amonia solution, and a mixture of 7% NaOH/ 12% urea/ 81 distilled water.

### SEM and TEM analyses

The size, morphology, and structure of the BNC samples were examined by a scanning electron microscope (SEM) and transmission electron microscope (TEM) (Supplementary Material).

### Energy-dispersive X-ray (EDX) spectroscopy

EDX analysis was performed using TEM “JEM-2100 Plus, JEOL Ltd., Japan; at the Central Laboratory, City of Scientific Research and Technological Applications, Alexandria, Egypt”.

### Thermogravimetric analysis (TGA)

TGA was performed using a TGA-50H Thermogravimetric analyzer on a sample of about 6 mg. The sample was scanned at a flow rate of 40 mL/min over a temperature range of ambient temperature to 800 °C.

### FTIR (Fourier transform infrared) spectroscopy analysis

FTIR spectroscopy analysis was conducted to analyze the surface properties of the BNC. The BNC sample was ground (crushed) with pure potassium bromide, and the mixture was pressed into a small tablet that was subjected to FTIR analysis^58^. The Shimadzu FTIR-8400 S spectrophotometer was used to measure the FTIR spectrum in the range of 4500 to 500 cm^−1^ at a resolution of 1 cm^−1^.

### Zeta potential analysis

Zeta potential of the cellulose sample was measured at “central Laboratories, City of Scientific Research and Technological Applications, Alexandria, Egypt” using a Malvern 3000 Zetasizer Nano ZS, UK”. The BNC suspension was diluted with deionized water to a concentration of 0.01 wt percent. Prior to the test, the diluted solution was homogenised in a high-speed homogenizer at a speed of 13,000 rpm for 10 min and then maintained in an ultrasonic bath. The sample was analyzed three times.

### X-ray Diffraction

X-ray Diffraction (XRD) was employed to evaluate the pattern and crystallinity degree of the BNC. The degree of crystallinity was determined using the empirical method proposed by Segal et al.^59^ equation from the diffracted intensity data (Supplementary Material).

## Results and discussion

### Screening of bacterial isolates for BNC production

**Figure 1:**
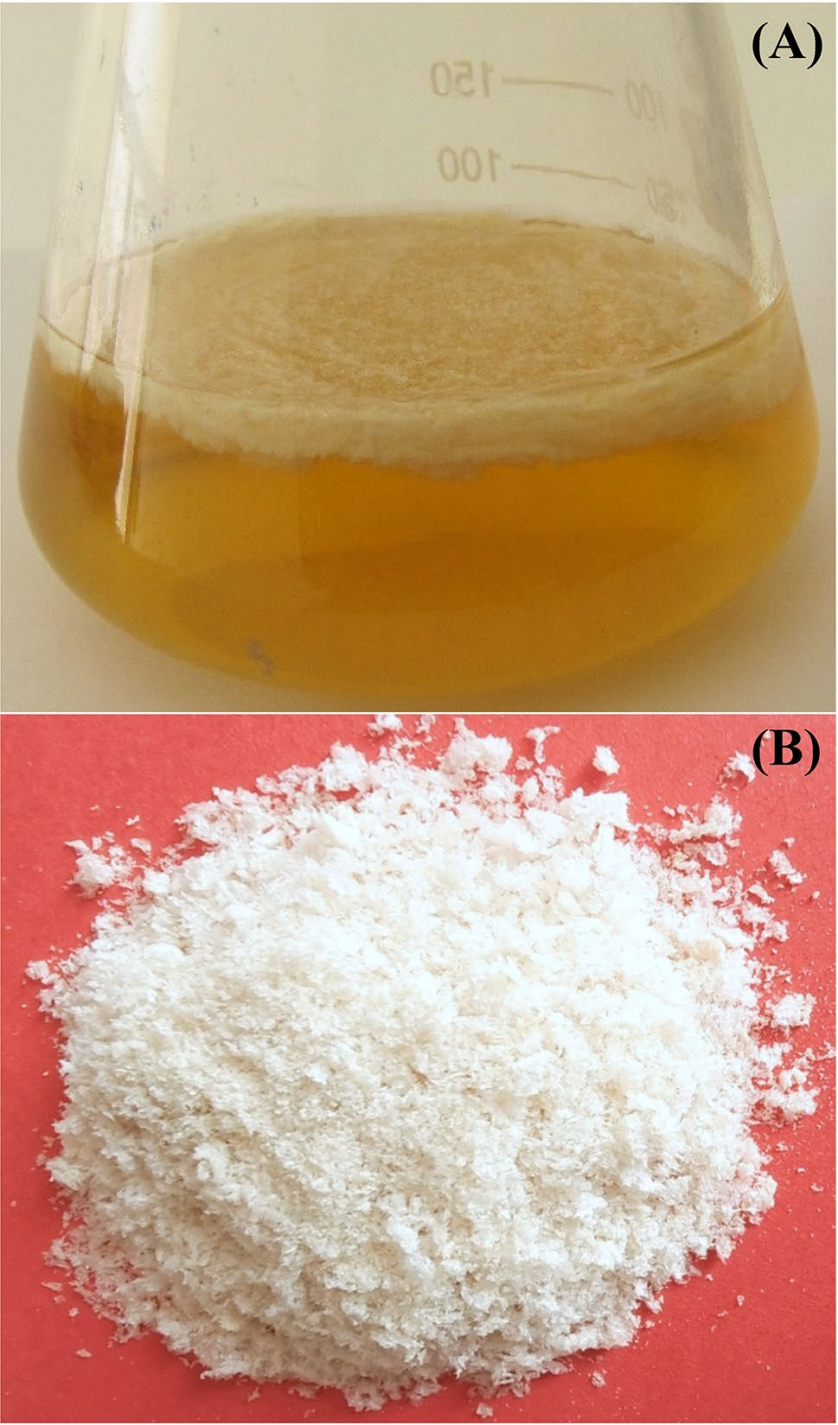
(**A**) Bacterial cellulose layer produced at the air–liquid interphase on medium no. 1; (**B**) Purified powder of the BNC.

A total of ten morphologically different bacterial strains were screened for their potential for BNC production. Among these isolates, four isolates exhibited the ability to produce the BNC. The most potent bacterial isolate (*Bacillus* sp*.* strain SEE-12) was selected for further BNC production and studies. After inoculation of the medium 1 and medium 2, the BNC layers were produced by *Bacillus* sp*.* strain SEE-12 through static fermentation at 30 °C for 7–14 days. As shown in Fig. 1A, the BNC was observed on the culture medium surface as a layer. The BNC was harvested, purified and the obtained purified white powder are shown in Fig. 1B. The maximum dry weights of the produced BNC were 10.5 and 9.75 g/L for medium 1 and medium 2 on day 10.

The fermentation process is normally carried out at around pH 4–7^60^. The pH of the medium strongly affects the BNC production and that the optimal pH for the BNC production is in the range of 4–6, as this is a favorable pH range for the bacteria^61^. Most studies have stated that there is a marked trend towards acidification, beginning with an acidic pH of 4.5 to 6, with a desired value of 5^62^. The culture medium with an initial pH of 4 was better for the BNC production using submerged cultivation^61^ and a pH of less than 4 is not suitable for the bacterial growth^60^.

### Identification of Bacillus sp. strain SEE-12 by taxonomic characteristics and 16S rRNA sequence analysis

**Figure 2:**
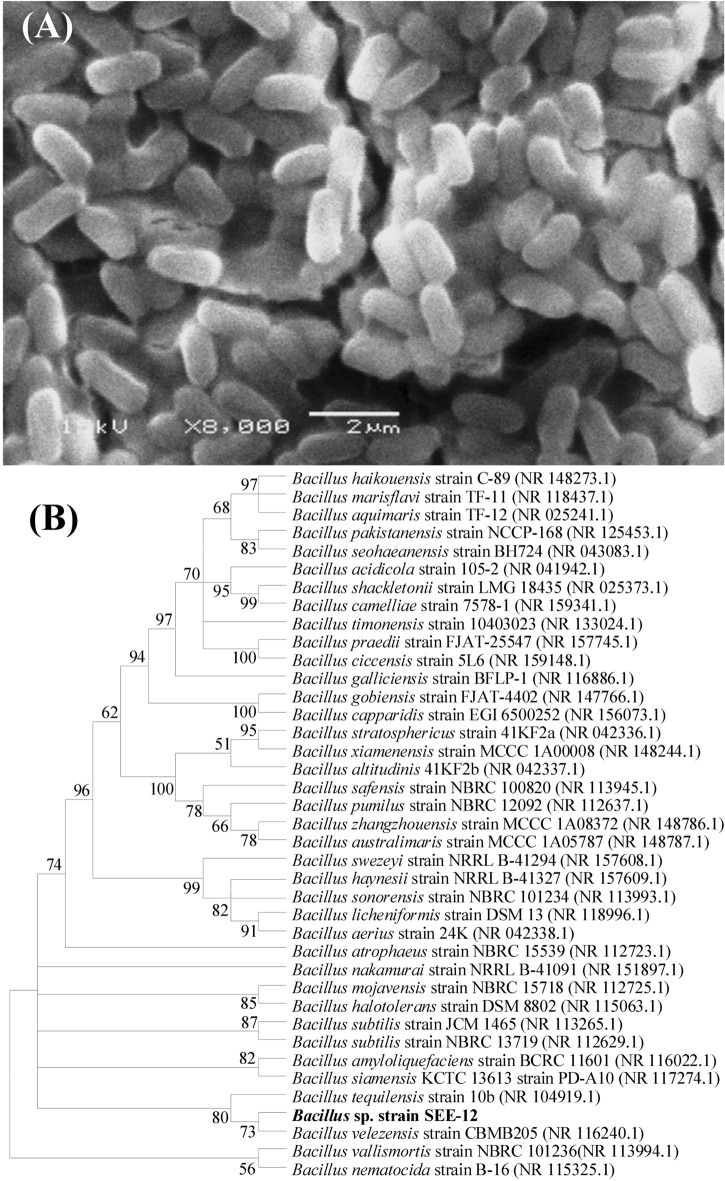
(**A**) Scanning electron micrograph showing cell morphology of strain SEE-12 at magnification of 8000 X, (**B**) The neighbor-joining algorithm phylogenetic tree of strain SEE-12 and related species of the genus *Bacillus*.

Supplementary Table S1 shows the main morphological and biochemical features of *Bacillus* sp*.* strain SEE-12. Colonies of *Bacillus* sp*.* strain SEE-12 are characteristically large and irregular in shape, with entire margins. They have a flat elevation and in surface growth in LB medium (Supplementary Figure S 1A). *Bacillus* sp*.* strain SEE-12 is an aerobic, Gram‐positive rods (Supplementary Figure S 1B). The microscopic examination indicated that *Bacillus* sp*.* strain SEE-12 is endospore‐forming rods, produces oval spores. Scanning electron microscopy has revealed rod-shaped bacilli (Fig. 2A). The strain is characterized by their capacity to utilize glucose, fructose, mannitol, glycine, starch, sucrose. While, ribose and xylose are not utilized by the strain *Bacillus* sp*.* strain SEE-12.

A pure culture of *Bacillus* sp*.* strain SEE-12 was identified by 16S rRNA sequencing. The obtained 16S rRNA fragment sequence of *Bacillus* sp*.* strain SEE-12 was amplified by polymerase chain reaction (PCR) using universal sequencing primers and the sequencing product was 1473 bp. The obtained 16S rRNA gene sequence was aligned with the released related 16S rRNA gene sequences available in the National Center for Biotechnology Information (NCBI) databases obtained using the BLASTN^63^. The phylogenetic tree (Fig. 2B) was generated by the use of MEGA version X software^52^ using the neighbour-joining analysis^64^. Only branches corresponding to bootstrap values above 50% bootstrap replicates are expressed. This analysis involved 39 nucleotide sequences. Based on the 16S rRNA gene sequence, phylogenetic analysis revealed that *Bacillus* sp*.* strain SEE-12 shared a high degree of similarity (98.58%) with *Bacillus tequilensis* strain 10b. Accordingly, *Bacillus* sp*.* strain SEE-12 was identified as *Bacillus tequilensis* strain SEE-12. The 16S rDNA gene sequence has been deposited under the accession number of MN826325 in the Gen Bank database.

### Effect of different carbon sources on the BNC production

The low productivity of the BNC is one of the industry's application obstacles. Nowadays, numerous carbon sources, including oligosaccharides, monosaccharides, organic acids, alcohol, have been used to enhance the BNC biosynthesis^49,65^. In this work, for the BNC production, the influence of different carbon sources and a low-cost carbon substrates on the BNC production by *Bacillus tequilensis* strain SEE-12 have been evaluated. A clear difference was observed for the BNC production from different carbon sources in the culture medium 1 and 2. The strain SEE-12 has a strong ability to produce the BNC using Cantaloupe juice, *Ulva lactuca* biomass extract, glycine, glucose, fructose and mannitol and a low ability to produce the BNC using sucrose. On the basis of dry weight of the BNC production (g/L medium), the highest production in the culture medium 1 was obtained on Cantaloupe juice (50%, v/v) followed by *Ulva lactuca* biomass extract, at 2% w/v carbon source concentration of glycine, glucose, fructose, mannitol and sucrose. The BNC production was 10.5, 7.73, 5.65, 4.79, 3.6, 3.55 and 1.92 g/L by using Cantaloupe juice, *Ulva lactuca* biomass extract, glycine, glucose, fructose, mannitol and sucrose; respectively (Supplementary Figure S2). The BNC was not produced on xylose, ribose and starch by *Bacillus tequilensis* strain SEE-12. Our findings are consistent with those of Castro et al.^66^, Embuscado et al.^67^, and others who have found that the type of carbon sources influences the formation of bacterial cellulose production in bacteria. Carbon sources are added to microbial fermentation media to promote the growth of the target microorganism, which leads to a higher rate of primary metabolite synthesis. Glucose, sucrose, and fructose have all been identified as suitable carbon sources for nanocellulose production. The highest amount (4 g/L) of BC by *Leifsonia* sp. CBNU-EW3 was achieved with glucose as carbon source^42^. The maximum cellulose yield (7.4 g/L) by *Rhodococcus* sp. MI 2 was achieved in SH medium containing 1.5% sucrose as carbon source^43^. The BC production by the *Lactobacillus hilgardii* IITRKH159 was evaluated with various carbon sources in Y-medium using one factor at a time approach. A high yield of BC was achieved using fructose followed by glycerol which was relatively higher than standard medium containing sucrose^29^.

Mohammadkazemi et al.^68^ reported that the highest production yield of bacterial cellulose using *Gluconacetobacter xylinus* strain PTCC 1734 was obtained using mannitol and sucrose as a carbon source which was greater than that achieved by utilizing glucose. Previous research by Ramana et al.^69^ demonstrated that sucrose, glucose, and mannitol were all appropriate for optimal cellulose production by *Acetobacter xylinum* and sucrose was found to be a more effective carbon source than syrup, glucose, or mannitol in terms of increasing the production of BNC. However, Embuscado et al.^67^ claimed that the production yield of bacterial cellulose in fructose-based medium is greater than that of sucrose. On the other hand, the highest yield of the bacterial cellulose production (3·83 g/L) by *Gluconacetobacter xylinus* strain ATCC 53,524 was obtained in a glucose-based culture medium^26^. Molina-Ramírez et al.^37^ found that the maximum yields of bacterial cellulose production by *Komagataeibacter Medellinensis* were 2.80, 0.38, and 1.68 g/L; respectively, when glucose, fructose, and sucrose were supplied at a concentration of 2% w/v. On the other hand, Ishihara et al.^65^ investigated the use of D-xylose as a carbon source for the bio synthesis of BNC and concluded that xylose is poorly assimilated by any strain of bacteria that capable of producing substantial amounts of BNC in glucose medium.

In the process of bacterial cellulose production, fruit juices were utilised as an alternative source of carbon^70^. Extracts made from the skins and peels of many fruits were also used as a culture medium for bacterial cellulose production^30^. These raw materials typically contain a significant amount of a variety of sugars, including sucrose, fructose, lactose, glucose and xylose^71^. Bagewadi et al.^38^ investigated the effect of various carbon substrates on BC production by *B. licheniformis* strain ZBT2 in HS medium. The glucose in the HS medium was substituted with 2%, w/v of five different carbon sources (fructose, maltose, sucrose, lactose, and galactose), and the effect of various low-cost carbon substrates on BC production by *B. licheniformis* strain ZBT2 was evaluated using apple juice, orange juice, grape juice, and sugarcane juice. They found that lactose is a better carbon source than glucose, and the highest production of BC was obtained by using HS medium supplemented with a low-cost carbon substrate, grape juice.

### Statistical screening of significant variables for the fermentation process affecting the BNC production by bacillus tequilensis strain SEE-12 using plackett–burman design

Plackett–Burman design was applied to determine the most significant variables for the maximum BNC production by *Bacillus tequilensis* strain SEE-12. In the present study, the influence of ten nutritional and environmental factors (namely A (peptone; g/L), B (yeast extract; g/L), C (Cantaloupe juice; %, v/v), D (Na_2_HPO_4_; g/L), E (citric acid; g/L), F (pH); G (medium volume; mL/250 mL conical flask), H (inoculum size; %, v/v), j (temperature; ºC), K (incubation time; days) in addition to one dummy variable) on the production of the BNC by *Bacillus tequilensis* strain SEE-12 through static fermentation was investigated (Supplementary Table S2). The experiment was conducted in 12 runs. The Plackett–Burman experimental design matrix selected for the screening of significant variables affecting the BNC production and the corresponding BNC production are shown in Supplementary Table S2. The BNC production varied markedly from 0.2 to 19.3 g/L (Supplementary Table S2). This variation reflected the importance of the process optimization to attain maximum production of the BNC. The results showed that the highest value of the BNC production (19.3 g/L) was obtained in the run no. 8 when the independent factors were: A (peptone; 5 g/L), B (yeast extract; 5 g/L), C (Cantaloupe juice; 50%, v/v), D (Na_2_HPO_4_; 5 g/L), E (citric acid; 1.5 g/L), F (pH 5); G (medium volume; 100 mL/250 mL conical flask), H (inoculum size; 5%, v/v), J (temperature; 37 °C), K (incubation time; 7 days) were used. While, the lowest value of the BNC production (0.2 g/L) was obtained in the run no. 4 when all the independent factors were adjusted to the lowest levels: A (peptone; 5 g/L), B (yeast extract; 5 g/L), C (Cantaloupe juice; 50%, v/v), D (Na_2_HPO_4_; 3 g/L), E (citric acid; 1.5 g/L), F (pH 3.6); G (medium volume; 50 mL/250 mL conical flask), H (inoculum size; 5%, v/v), J (temperature; 30 °C), K (incubation time; 4 days).

### Statistical analysis of plackett–burman design for the BNC production by bacillus tequilensis strain SEE-12

**Figure 3:**
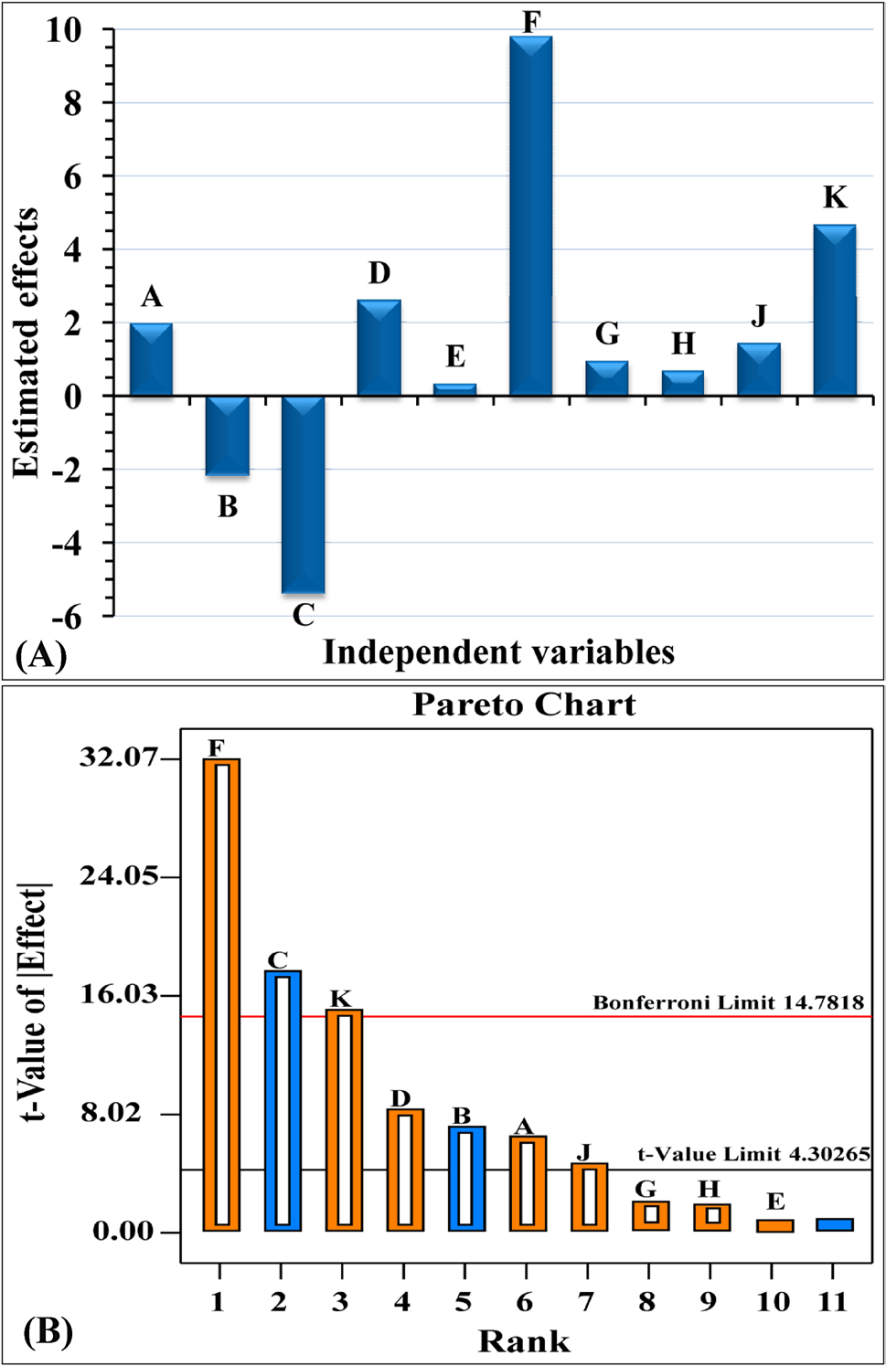
**(A**) Estimated effects of independent factors on BNC production by *Bacillus tequilensis* strain SEE-12 using Plackett–Burman design, (**B**) Pareto chart shows the order and significance of the factors.

The relationship between the BNC production by *Bacillus tequilensis* strain SEE-12 and the independent variables was analyzed by multiple-regression statistical analysis of the Plackett–Burman design results, and the analysis of variance (ANOVA) was calculated and presented in Table 1. Table 1 and Fig. 3A show the estimated effect of each independent factor on the BNC production. A high estimated effect, either positive or negative, means that the independent variable has a significant effect on the response. If the estimated effect of a certain tested variable has a positive sign, this means that production is increased at a high level of the variable. On the other hand, when the sign is negative, it indicates that production is higher when the variable is at a low value^72^. The contribution percentages of each variable are also given in Table 1. The regression coefficients for the ten nutritional and environmental factors (Table 1) show how much each factor affects the production of the BNC. The signs of the coefficients and effects were used to interpret the data^73^ (positive or negative effects on the BNC production). It was clear that eight of the ten factors, including: peptone, Na_2_HPO_4_, citric acid, pH, medium volume, inoculum size, temperature and incubation time with coefficient value 0.98, 1.29, 0.16, 4.90, 0.47, 0.33, 0.71 and 2.33 and percent of contribution 2.33, 4.05, 0.06, 57.98, 0.53, 0.26, 1.20 and 13.09; respectively had positive effects on the production of the BNC (Table 1 and Fig. 3A) which means that the increase in their levels could exert positive effect on the production of the BNC. Among the independent factors, yeast extract (B) and Cantaloupe juice (C) with coefficient value − 1.09 and − 2.70 and percent of contribution 2.85 and 17.59%; respectively, both exhibited negative impacts, suggesting that decreasing the concentrations of yeast extract and Cantaloupe juice could have a favorable effect on the production of BNC.

**Table 1:** Statistical analysis of Plackett–Burman design showing coefficient estimate, effect, % contribution, *F*-value and *P*-value for each factor affecting BNC production.

Term	*df*	Coefficient estimate	Effect	% Contribution	*F*-value	*P*-value
Model	9	7.44			196.84	0.0051*
A-Peptone	1	0.98	1.96	2.33	41.24	0.0234*
B-Yeast extract	1	− 1.09	− 2.17	2.85	50.55	0.0192*
C-Cantaloupe juice	1	− 2.70	− 5.40	17.59	311.95	0.0032*
D-Na_2_HPO_4_	1	1.29	2.59	4.05	71.80	0.0136*
E-Citric acid	1	0.16	0.32	0.06	1.24	0.4652
F-pH	1	4.90	9.80	57.98	1028.29	0.0010*
G-Medium volume	1	0.47	0.94	0.53	9.44	0.0916
H-Inoculum size	1	0.33	0.66	0.26	4.69	0.1627
J-Temperature	1	0.71	1.41	1.20	21.36	0.0438*
K-Incubation time	1	2.33	4.66	13.09	232.24	0.0043*
Std. Dev	0.53	R-Squared	0.9989			
Mean	7.44	Adj R-Squared	0.9938			
C.V.%	7.11	Pred R-Squared	0.9594			
PRESS	20.15	Adeq Precision	40.14			

* Significant values, *F* Fishers's function; *P* Level of significance; *C.V* Coefficient of variation.

The Pareto chart illustrates the importance ranking of the factors affecting nanocellulose biosynthesis (Fig. 3B). Pareto chart illustrates the relationship between *t*-value (the absolute values of the effects) and ranks. In the Plackett–Burman experimental design, the Pareto chart is used to determine the order of the relative importance of the variables influencing the response. Any effect that extends beyond the chart's reference line is highly significant. Pareto chart (Fig. 3B) showed that pH (F) was the most significant independent factor affecting the production of the BNC by *Bacillus tequilensis* strain SEE-12; followed by Cantaloupe juice (C) and incubation time (K) with probability values of 0.0010, 0.0032 and 0.0043; respectively and have been chosen for central composite design optimization.

The determination coefficient (*R*^2^) was used to verify the model's fit. The *R*^2^ value indicates the extent to which the experimental parameters can explain the observed response values. The value of *R*^2^ is always in the range of 0 to 1. When the determination coefficient (*R*^2^) value is close to 1, the design is more precise for predicting the response^74^. In the present study, the *R*^2^ value is 0.9989 indicating that the model is fit and able to provide an explanation of 99.98% of the variability in the BNC production by *Bacillus tequilensis* strain SEE-12. The model cannot explain only 0.02 percent of the overall variability in BNC synthesis by *Bacillus tequilensis* strain SEE-12. Additionally, the adjusted determination coefficient's value (Adj. *R*^2^ = 0.9938) is also extremely high and emphasized that the model is highly significant (Table 1). The higher values of the *R*^2^ and *R*^2^ adj refer to a highly significant model that is suitable for explaining the relationships between the experimental variables that have been chosen and the BNC production by *Bacillus tequilensis* strain SEE-12. The predicted-*R*^2^ indicates how well the model predicts responses for new experiments, thus our results revealed that this model is capable of accounting for the value of nanocellulose production with 95.94% accuracy in the range of the factors tested. Likewise, the predicted-*R*^2^ value of 0.9594 for the BNC production by *Bacillus tequilensis* strain SEE-12 is high and agrees reasonably well with the adjusted-*R*^2^ value of 0.9938 which confirm the model's statistical validity and reliability. The predicted-*R*^2^ and Adjusted-*R*^2^ values must be within 20% of each other, so that we can say that there is a high significance and accuracy of the model and also there is a reasonable agreement between them^75^.

*P* and *F*-values were calculated to determine the importance of the model and the variables (Table 1). The ANOVA of the Plackett–Burman design indicated that the model is highly significant as it is evident from a very low probability value (*P*-value = 0.0051) and the *F*-value of 196.84. Also, the process factors with *P*-values less than or equal to 0.05 (confidence levels greater than or equal to 95 percent) were considered to have significant impacts^76^. In the current study, the results revealed that peptone, yeast extract, Cantaloupe juice, Na_2_HPO_4_, pH, medium volume, temperature and incubation time are eight significant variables (*P* < 0.05) for the BNC production. The results showed that, pH (*P*-value 0.0010 and *F*-value 1028.29) was the most significant independent factor affecting the production of the BNC by *Bacillus tequilensis* strain SEE-12; followed by Cantaloupe juice (*F*-value 311.95 and *P*-value 0.0032), and incubation time (*F*-value 232.24 and *P*-value 0.0043). The data revealed that, citric acid (E) and inoculum size (H) are two non-significant independent factors (*P*˃0.05) with lower effects (0.32 and 0.66; respectively) and lower percent of contribution (0.06 and 0.26; respectively).

The adequate precision value measures the signal to noise ratio. A value higher than 4 is desirable and indicates the good fit of the model^77^. The adequate precision value of the present model is 40.14 and this value suggests that the model can be used to navigate the design space. The model showed PRESS, mean, standard deviation and C.V.% (coefficient of variation) values of 20.15, 7.44, 0.53 and 7.11; respectively (Table 1).

The regression coefficients were calculated (Table 1) and the data was fitted to the first-order polynomial equation to describe the relationship between the independent factors and the BNC production by *Bacillus tequilensis* strain SEE-12. The equation of the model fitted with a regression analysis was obtained in terms of the coded independent factors:

1 $$\begin{aligned} Y \, & = + 7.44 + 0.98{\text{A}} - 1.09{\text{ B}} - 2.70{\text{ C}} + 1.29 \, D + 0.16{\text{ E}} \\ & \quad + 4.90{\text{ F}} + 0.47{\text{ G}} + 0.33{\text{ H}} + 0.71{\text{ J}} + 2.33{\text{ K}} \\ \end{aligned}$$

where Y is the BNC production, and the independent factors are A (peptone), B (yeast extract), C (Cantaloupe juice), D (Na_2_HPO_4_), E (citric acid), F (pH); G (medium volume), H (inoculum size), J (temperature) and K (incubation time).

In a confirmation experiment, to determine the precision of Plackett–Burman, the parameters thought to be optimal for maximum production of the BNC by *Bacillus tequilensis* strain SEE-12 were A (peptone; 5 g/L), B (yeast extract; 5 g/L), C (Cantaloupe juice; 50%, v/v), D (Na_2_HPO_4_; 5 g/L), E (citric acid; 1.5 g/L), F (pH 5); G (medium volume; 100 mL/250 mL conical flask), H (inoculum size; 5%, v/v), J (temperature; 37 °C), K (incubation time; 7 days) were used. Under these conditions, the maximum production of the BNC was 19.3 g/L which is higher than the BNC gained prior to the use of Plackett Burman (10.5 g/L) by 1.84 times.

### Model adequacy checking

**Figure 4:**
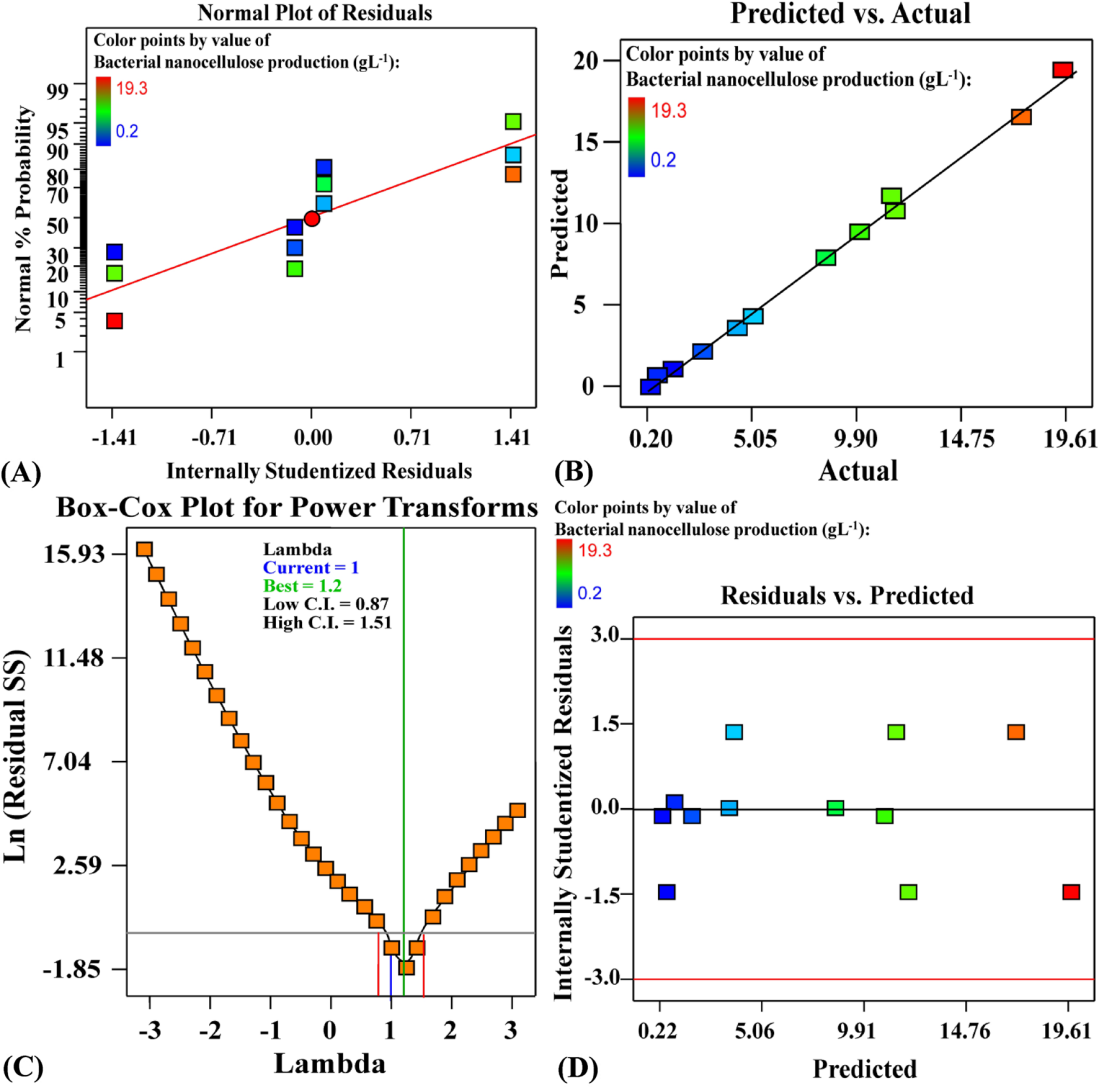
**(A**) Normal probability plot of internally studentized residuals; (**B**) plot of predicted versus actual; (**C**) Box-Cox plot of model transformation and; (**D**) plot of internally studentized residuals versus predicted values of BNC production by *Bacillus tequilensis* strain SEE-12 using Plackett–Burman design.

The normal probability plot (NPP) of the residuals is a critical graphical approach for visualising the residuals' distribution and determining the model's adequacy^78^. The residuals are the difference between the theoretical model's predicted response values and the experimental response values. A little residual value suggests that the model prediction is highly accurate and that the model fits the experimental results well. Figure 4A plots the NPP of the studentized residuals against the model's predicted response values. The residuals points in this study are normal distributed; they are located next to the diagonal straight line, indicating the model's validity with the experimental results of the BNC production. Deviations from this straight line indicate that the residuals are not normally distributed.

Figure 4B depicts a plot of predicted versus actual BNC production, with points near to the fitted line, indicating a significant correlation among the model's predicted values and the experimental results of the BNC production which confirms the accuracy of the model^79^. The Box–Cox graph of model transformation of the BNC production is shown in Fig. 4C. As can be seen in Fig. 4C, the green line representing the best lambda value (Lambda (λ) = 1.2) and the blue line representing the current transformation optimal value (λ = 1). The current transformation confidence interval value (λ = 1) is quite near to the best value (best = 1.2), the model is well fit to the obtained experimental results. Therefore, the model is in the perfect zone, as the blue line of the current transformation (λ = 1) lies between the two vertical red lines (minimum and maximum values of confidence intervals between 0.87 and 1.51, respectively) indicating that the model fits the experimental data well and no data transformation is needed. A plot of the predicted BNC production vs the studentized residuals is shown in Fig. 4D. The residuals were distributed uniformly and randomly above and below the zero line, with no distinct pattern, demonstrating that the residuals have a constant variance and validating the model's accuracy.

### Statistical optimization of fermentation process variables for the BNC production by bacillus tequilensis strain SEE-12 using face centered central composite design (FCCCD)

Based on the effects and *P-*values (Table 1), pH (X_1_), Cantaloupe juice concentration (X_2_), and incubation time (X_3_), were selected for further optimization using FCCCD (Face centered central composite design), as these were the most significant independent variables affecting the BNC production. Supplementary Table S3 shows the FCCCD design matrix used to optimize the three most significant independent process variables affecting the BNC production and their concentrations at various coded and actual levels. Other variables were set at their optimal points of Plackett–Burman design. The experimental and predicted BNC production for the 20 runs of the Face centered central composite design are shown in Supplementary Table S3, which demonstrates significant variance in the quantity of BNC produced depending on the levels of the fermentation process variables. Based on the experimental data obtained; the BNC production ranged from 6.40 to 22.80 g/L of medium, the highest dry weight of the BNC production was obtained in the run no. 13 (center point) with value of 22.80 g/L of the medium, where initial pH value was 5, Cantaloupe juice (%, v/v) was 50 mL and incubation time was 6 days, while the minimum BNC production was obtained in the run no. 4 with value of 6.40 g/L of medium, where initial pH value was 4, Cantaloupe juice (%, v/v) was 25 mL and incubation time was 4 days.

To better understand the efficacy of *Bacillus tequilensis* strain SEE-12 in the production of BC, a comparison was made with BC production yield of Gram-positive and Gram-negative BC-producing bacteria in the previous reports.

In the previous studies, the maximum cellulose production with Gram-positive bacterial strains ranged between 4 and 9.2 g/L. These values are less than the bacterial nanocellulose yield attained by *Bacillus tequilensis* strain SEE-12 in the current study, which was 22.80 g/L. The BC production yield by *Bacillus tequilensis* strain SEE-12 was 2.47 times higher than BC yield by *Bacillus licheniformis* strain ZBT2, which was 9.2 g/L^38^, 3.08 times higher than BC yield by *Rhodococcus* sp. MI 2, which was 7.4 g/L of cellulose^43^, 3.15 times higher than BC yield by *Lactobacillus hilgardii* IITRKH159, which was 7.23 ± 0.59 g/L^29^, 3.45 times higher than BC yield by *Bacillus amyloliquefaciens* ZF-7, which was 6.6 g/L of cellulose^39^, 3.81 times higher than BC yield by *Leifsonia* sp., which was 5.97 g/L^41^, 5.42 times higher than BC yield by *Lactobacillus mali* cocultured with *Gluconacetobacter xylinus*, which was 4.2 g/L^80^, 5.70 times higher than BC yield by *Leifsonia* sp. CBNU-EW3, which was 4 g/L of cellulose^42^.

In the previous studies, the maximum cellulose production with Gram-negative BC-producing bacteria ranged between 2.8 and 18.68 g/L. The BC production yield by *Bacillus tequilensis* strain SEE-12 was 1.22 times higher than BC yield by a kombucha consortium (*Brettanomyces anomalus* KY103303*, Brettanomyces bruxellensis* MH393498 and *Komagataeibacter saccharivorans* LN886705), which was 18.68 g/L^81^, 2 times higher than BC yield by *Komagataeibacter xylinus* IITR DKH20, which was 11.4 g/L^29^, 2.59 times higher than BC yield by *Acetobacter xylinus* NBRC 13,693, which was 8.8 g/L^30^, 3.04 times higher than BC yield by *Komagataeibacter xylinus* BPR 2001, which was 7.5 ± 0.54 g/L^31^, 3.2 times higher than BC yield by *Gluconoacetobacter hansenii* NCIM 2529, which was 7.129 g/L^32^, 3.89 times higher than BC yield by *Komagataeibacter rhaeticus* K3, which was 5.85 g/L^33^, 4.01 times higher than BC yield by *Komagataeibacter xylinus* ATCC 700,178, which was 5.69 g/L^17^, 4.83 times higher than BC yield by *Gluconacetobacter xylinum* ATCC, which was 4.72 g/L^34^, 5.95 times higher than BC yield by *Gluconacetobacter xylinus* strain ATCC 53,524, which was 3.83 g/L^26^, 6.82 times higher than BC yield by *Gluconacetobacter xylinus* strain, which was 3.34 g/L^35^, 7.13 times higher than BC yield by *Gluconacetobacter xylinus*, which was 3.2 g/L^36^, 8.14 times higher than BC yield by *Komagataeibacter Medellinensis*, which was 2.80 g/L^37^.

The variations in the yield of BC produced by different bacterial strains may be attributable to several factors, including but not limited to the composition of fermentation medium especially carbon and nitrogen sources supplied in the medium, fermentation conditions (fermentation time, pH, temperature, agitation or static), the producing strain types^29^ and the approaches used to maximize the BC production.

### Multiple regression analysis and ANOVA

Using design expert 11 software, the FCCCD experimental data was analyzed using multiple regression analysis. Tables 2, Supplementary Table S4 demonstrate multiple regression analysis and analysis of variance (ANOVA). A regression model with an *R*^2^-value greater than 0.9 is considered to have an extremely high degree of correlation^82^. In this model, the *R*^2^ value = 0.9945 (Table 2) indicated that 99.45% of the BNC production variations can be described by the selected model and the selected independent variables, and only 0.55 percent of the overall variation is unexplained by the model. According to Koocheki et al.^83^, a high *R*^2^ value does not always reflect a good regression model, and such an assumption can be made only if the adjusted *R*^2^ value (adjusted determination coefficient) is comparatively high. The adjusted *R*^2^ value was found to be 0.9895 which is extremely high suggesting that the model's fit to the experimental data is highly significant. It demonstrated a strong match between the predicted values and the experimental values of the BNC production. Predicted *R*^2^ is a statistical measure of the model's adequacy for predictions of the response values in the range of the experimental variables. The ‘predicted *R*^2^’ value of 0.9859 is also high indicating the high significance of the model for prediction of the BNC production (Table 2). The predicted *R*^2^ value (0.9859) is consistent with the value of the adjusted *R*^2^ value (0.9895) revealing a high degree of agreement between all experimental and predicted values for BNC biosynthesis.

**Table 2:** Statistical analysis for FCCCD of BNC production.

Source of variance		Sum of squares	*df*	Mean square	*F-*value	*P-*value *P*rob > *F*	Coefficient estimate
Model		485.97	9	54.00	199.52	< 0.0001*	21.77
Linear effects	X_1_	11.32	1	11.32	41.83	< 0.0001*	1.06
	X_2_	4.04	1	4.04	14.95	0.0031*	− 0.64
	X_3_	18.06	1	18.06	66.75	< 0.0001*	1.34
Interactioneffects	X_1_ X_2_	4.26	1	4.26	15.75	0.0026*	− 0.73
	X_1_ X_3_	10.22	1	10.22	37.75	0.0001*	− 1.13
	X_2_ X_3_	10.22	1	10.22	37.75	0.0001*	− 1.13
Quadratic effects	X_1_^2^	40.40	1	40.40	149.27	< 0.0001*	− 3.83
	X_2_^2^	29.82	1	29.82	110.17	< 0.0001*	− 3.29
	X_3_^2^	37.09	1	37.09	137.07	< 0.0001*	− 3.67
Error effects	Lack of Fit	0.35	5	0.07	0.15	0.9713	
	Pure Error	2.35	5	0.47			
**Std. Dev.**	0.52	**R**^**2**^	0.9945				
**Mean**	16.37	**Adj R**^**2**^	0.9895				
**C.V.%**	3.18	**Pred R**^**2**^	0.9859				
**PRESS**	6.91	**Adeq Precision**	42.30				

The independent factors are: X_1_ (pH); X_2_ (Cantaloupe juice; %, v/v); X_3_ (incubation time; days).

*Significant values *F* Fishers's function; *P* Level of significance; *C*.*V* Coefficient of variation.

The negative coefficient values denote an antagonistic relationship between the linear, mutual interactions or quadratic effects of the variables and the BNC production by *Bacillus tequilensis* strain SEE-12 (the variables negatively affect the BNC production), whereas the positive coefficient values indicate a synergistic relationship between the variables and the BNC production by *Bacillus tequilensis* strain SEE-12 in the tested range of these variables (Table 2). The ANOVA of the quadratic regression model indicates goodness of fit of the model and the Fisher’s *F* test revealed that the model terms are significant (*F-*value = 199.52) with a very low *P-*value (< 0.0001) and a lack of fit that is not statistically significant (*P*-value = 0.9713) (Table 2). Furthermore, the coefficient of variation value (CV% = 3.18%) is relatively low indicates a better precision and reliability of the model (Table 2). The predicted value for the PRESS (residual sum of squares) is 6.91, the adequate precision value is 42.30, standard deviation value is 0.52 and the mean value of the present model is 16.37 (Table 2). Therefore, non-significance lack-of-fit, the high adjusted *R*^2^ value of the model, low PRESS value, low coefficient of variance, low standard deviation, high adequate precision and high *F*-value demonstrated the high accuracy and reliability of the model used in predicting the BNC production by *Bacillus tequilensis* strain SEE-12. Furthermore, it is obvious from the *P*-values of the coefficients that all linear coefficients, all interactions effects between X_1_, X_2_ and X_3_, quadratic effect of X_1_, X_2_ and X_3_ had a very significant effect and contribute to the BNC production by *Bacillus tequilensis* strain SEE-12 (Table 2).

The fit summary results contributed to select the proper model that fit the FCCCD used for the BNC production by *Bacillus tequilensis* strain SEE-12. The quadratic model type (Supplementary Table S4) with non significant lack of fit (*P-*value = 0.9713) and a very low *P-*value (< 0.0001) is the adequate model fitting the BNC production by *Bacillus tequilensis* strain SEE-12. Also, the quadratic model summary statistics showed the lower standard deviation of 0.52, the lower PRESS value of 6.91 and the higher R-squared value (0.9945) adjusted R-squared value (0.9895) and predicted R-squared value (0.9859).

The regression coefficients have been estimated and fitted to a polynomial equation of the second-order. The BNC production (Y) by *Bacillus tequilensis* strain SEE-12 can be predicted by the following regression equation:

6 $$\begin{aligned} Y \, & = + \, 21.77 \, + \, 1.06{\text{ X}}_{1} - 0.64{\text{ X}}_{2} + \, 1.34{\text{ X}}_{3\,} - \,0.73 \, X_{1} X_{2\,} \\ & \quad - \,1.13{\text{X}}_{1} {\text{X}}_{3\,} - \,1.13{\text{ X}}_{2} {\text{X}}_{3\,} - \,3.83{\text{ X}}_{1}^{2\,} - \,3.29{\text{ X}}_{2}^{2\,} - \,3.67{\text{ X}}_{3}^{2} \\ \end{aligned}$$

where Y was the predicted BNC production and the coded levels of independent factors were: X_1_ (pH); X_2_ (Cantaloupe juice; %, v/v); X_3_ (incubation time; days).

### Contour and three dimensional (3D) plots

**Figure 5:**
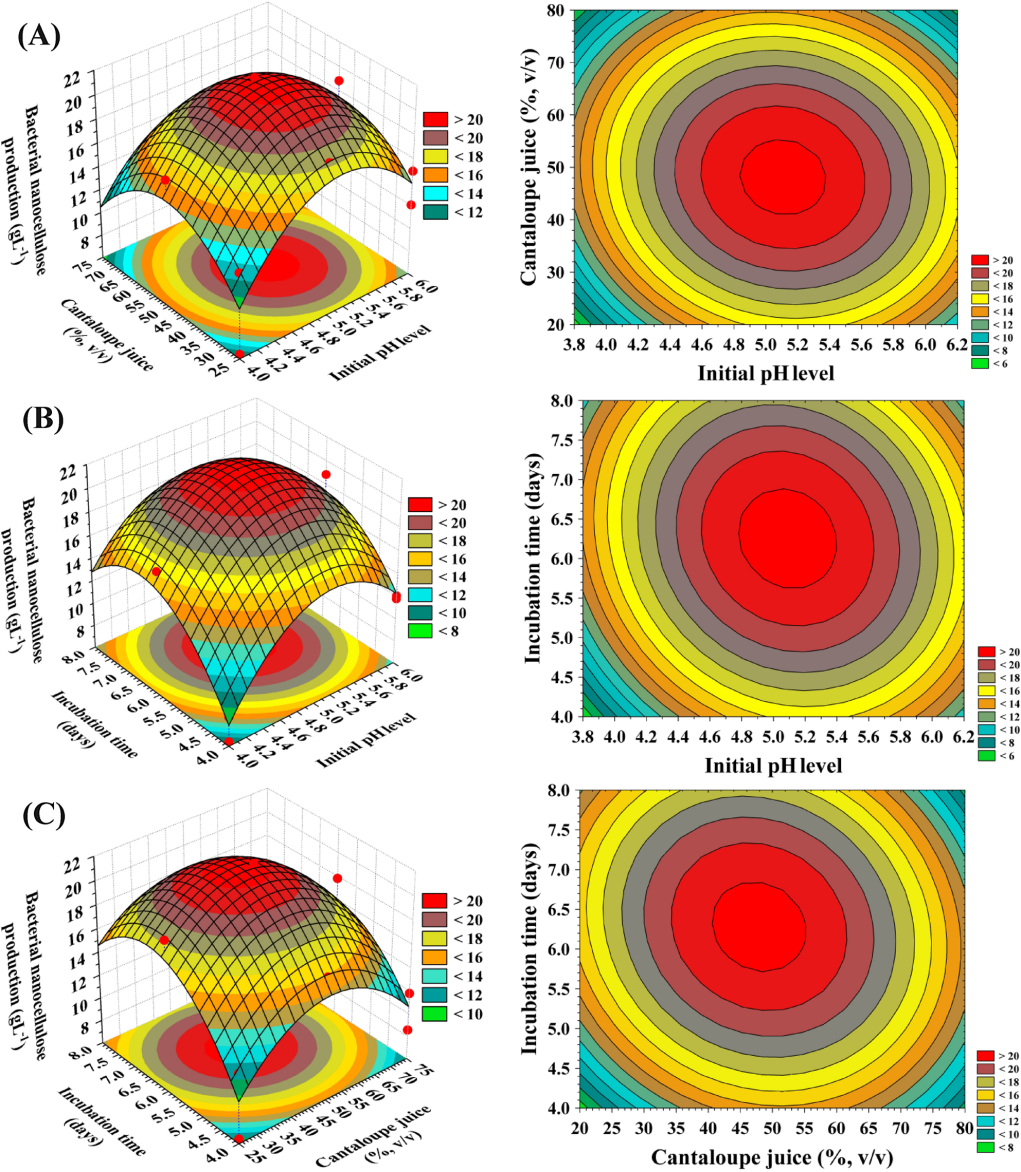
(**A–C**). 3D plots showing the mutual effects of pH (X_1_), Cantaloupe juice (X_2_) and incubation time (X_3_) on BNC production.

The 3D and contour graphics (Fig. 5A–C) provides a view of the correlation between the BNC production and the interactions between test variables in order to identify the optimal conditions for the production of BNC. For the pair-wise combinations of the three significant variables, three-dimensional graphs were generated (X_1_ X_2_, X_1_ X_3_ and X_2_ X_3_) by drawing the Z-axis response (the BNC production) against X and Y-axes for independent factors while fixing the value of the other variable at zero (center points).

The three-dimensional and contour graphs (Fig. 5A) demonstrating the reciprocal interactions of the initial pH (X_1_) and Cantaloupe juice (X_2_) on the BNC production, while the incubation time (X_3_) was maintained at the central level (6 days). Figure 5A demonstrates that decreased levels of both initial pH (X_1_) and Cantaloupe juice (X_2_) support low BNC production. The maximum BNC production very close to the middle levels of both initial pH (X_1_) and Cantaloupe juice (X_2_). On the other hand, further increase in levels of both initial pH (X_1_) and Cantaloupe juice concentration (X_2_) resulted in a gradual decrease in the BNC production.

Previously, Tahara et al.^84^ studied the effect of pH on the production of the BNC. They reported that in a constant pH medium of 5, the BNC production was higher than in a medium with pH 4. Al-Abdallah and Dahman^28^ reported that a pH of 2.0 is considered to be a suitable pH when *G. xylinus* ATCC 700,178 and wheat straw are used as the growth medium. By contrast, Kiziltas et al.^85^ studied the pH effect on the BNC production; they found that an alkaline pH 8 was the optimum pH for the cultivation of *A. xylinus* 23,769 at an incubation temperature of 28 °C in hot water extracted-wood. However, Castro et al.^66^ isolated *Gluconacetobacter medellensis* with the ability to produce high amounts of the bacterial cellulose at a highly acidic pH, 3.5. Subsequently, Urbina et al.^60^ reported that *Gluconacetobacter medellensis* cell viability was favoured in low pH media. The optimum initial pH for the bacterial cellulose production by cellulose producing bacterial strain, *Gluconacetobacter* sp. gel_SEA623-2, was 3.5^86^. The best performance was achieved by the DHU-ATCC-1 strain when it produced bacterial cellulose in a medium with an initial pH of 4 via submerged cultivation^61^. Velásquez-Riaño and Bojacá^62^ reported that for a maximum bacterial cellulose production in a low-cost medium with bacteria of the *Komagataeibacter* genus was obtained using 1–2% of the initial concentration of sugar at 28–30 °C of the temperature and there is no need for the initial pH be constant (between 4.5 and 5.5), the optimal incubation time was 7 days.

Fruits are distinguished by a high concentration of carbohydrates such as glucose and fructose and a low pH, which enables them to be used (particularly juices and extracts) to culture microorganisms such as the acetic bacteria that produce cellulose^62^. The Cantaloupe fruits have been processed into clarified juice by squeezing the frozen and thawed Cantaloupe flesh in a blender with the peel removed and then filtered. The Cantaloupe juice pH was 6.52, the sugar composition values were (g/100 mL): sucrose 1.73 , glucose 1.23 and fructose 1.61^87^. The maximum BNC production by *Komagataeibacter xylinus* IITR DKH20 with extract of pineapple peel waste was found as 11.4 g/L^16^. Meanwhile, Güzel and Akpınar^15^ have reported that the highest BNC production by *Komagataeibacter hansenii* GA2016 was 1.54% and 11.53% for apple peel and kiwifruit hydrolysates; respectively. On the other hand, Kurosumi et al.^30^ reported that the maximum BNC production with *Acetobacter xylinus* NBRC 13,693 was 8.8 and 6.9 g/L using a fruit juice like grape and orange juice; respectively in HS medium.

The 3D and contour graphics (Fig. 5B) describes the effects of the initial pH value (X_1_) and incubation time (X_3_) on the BNC production, while Cantaloupe juice concentration (X_2_) was kept at its zero level (50%, v/v). It can be seen that, the BNC production increases gradually by increasing the incubation time till reach its optimum after about 6 days of incubation, after which the BNC production decreased. As the value of the initial pH (X_1_) increased, the BNC production increased gradually up to pH 5 and then the BNC production decreased with further increase in the initial pH.

In the current study, the maximum yield of the BNC production by *Bacillus tequilensis* strain SEE-12 was obtained after about 6 days of incubation. In addition, when compared to other BC-producing strains, *Bacillus tequilensis* strain SEE-12 is more effective in producing BNC at a high yield (22.8 g/L) while requiring less incubation time. Nguyen et al.^35^ have reported that cellulose production with *a Gluconacetobacter xylinus* strain was 3.34 g/L after 7 days of incubation. On the other hand, the bacterial cellulose production of *Gluconacetobacter xylinus* AS6 was determined as 0.251 ± 0.03 g/L and the incubation was lasted for a week^88^. While, the maximum yield of the bacterial cellulose production (6.32 g/L) by *Komagataeibar xylinus* DSMZ 2004^89^ and *Komagataeibacter xylinus* IITRDKH20^16^ was obtained after 16 days. However, the maximum yield of the bacterial cellulose production by *Komagataeibacter rhaeticus* SU12 (25.34 g/L) was achieved after 21 days^90^.

The 3D and contour graphics (Fig. 5C) highlight the roles played by Cantaloupe juice concentration (X_2_) and incubation time (X_3_) on the BNC production when the initial pH (X_1_) was kept at its central level (pH = 5). As the Cantaloupe juice concentration (X_2_) and incubation time (X_3_) increased, the BNC production increased gradually up to their optimum levels and thereafter the BNC production decreased.

### Desirability function (DF)

To identify the optimal predicted conditions for maximal response, the desire function (DF) was applied. DF values ranged from 0 (undesirable) to 1 (desirable). The desirability function value is often determined mathematically prior to experimental validation of the optimization process^91^. The optimal predicted conditions for maximal response were identified using the DF option in the Design Expert Software (version 11). The optimization plot in Supplementary Figure S3 illustrates the desirability function and the predicted optimum values for maximum BNC production. In order to verify the BNC production under the optimal predicted conditions, an experiment was conducted in triplicate using the optimized growth conditions and the obtained experimental BNC (22.8 g/L) was compared to the model-predicted BNC production (22.01 g/L). The verification showed that the experimental and predicted values of the BNC production are in excellent agreement imply that the DF accurately predicts the optimal predicted values for maximum BNC biosynthesis.

A comparison between *Bacillus tequilensis* strain SEE-12 and some BC producers in terms of BC production and optimum conditions was shown Table 3.^17,29,31,35,38,39,41–43,81,88^.

**Table 3:** A comparison between *Bacillus tequilensis* strain SEE-12 and some BC producers in terms of BC production and optimum conditions.

Organism	Optimum conditions	BC production (g/L)	References
*Bacillus tequilensis* strain SEE-12	g/L: 5 peptone, 5 yeast extract, 1.5 citric acid, 5 Na_2_HPO_4_ by using 50%, v/v Cantaloupe juice, pH 5, medium volume of 100 mL/250 mL conical flask, inoculum size 5%, v/v at 37 °C for 6 days under static conditions	22.8	Current study
A kombucha consortium	6% glucose and1% black tea as nitrogen source, pH 6 at 30 °C for 10 days under static conditions	18.68	Avcioglu et al. ^81^
*Komagataeibacter xylinus* IITR DKH20	70% (v/v) extract of pineapple peel waste, after 16 days and pH 6	11.4	Khan et al.^15^
*B. licheniformis* strain ZBT2	HS medium supplemented with grape juice (5%, v/v), yeast extract (1.5% w/v) and peptone (1.5% w/v)	9.2	Bagewadi et al.^38^
*Komagataeibacter xylinus* BPR 2001	% (m/v): citric acid 0.115, Na_2_HPO_4_ 0.27, (NH_4_)_2_SO_4_ 0.63, corn steep liquor (CSL) 1.91, molasses 5.38 and ethanol 1.38% (v/v), pH 5.5 after 9 days at 30 °C under static conditions	7.5 ± 0.54	Rodrigues et al.^31^
*Rhodococcus* sp. MI 2	HS medium containing 1.5% sucrose, 0.9% yeast extract, 0.7% peptone and 5% (v/v) inoculum at pH 3.5 and 25 °C under static conditions after14 days	7.4	Tanskul et al.^43^
*Lactobacillus hilgardii* IITRKH159	High yield of BC was recorded from 50 g/L fructose	7.23 ± 0.59	Khan et al. ^29^
*B. amyloliquefaciens* ZF-7	High yield of BC was recorded in the culture medium under static conditions	6.2	Zhu et al.^39^
*Leifsonia* sp.	HS medium supplemented with maltose at 2% (w/v) and 5 mL/L of soy whey as nitrogen source, calcium chloride at a concentration of 50 mM, pH 6.5 at 30 °C after 7 days when the inoculum size is 10%	5.97	Rastogi and Banerjee^41^
*Komagataeibacter xylinus* ATCC 700,178	1.26 g/L citric acid and 3.39 g/L Na_2_HPO_4_, 1.5% m/v recycled paper sludge hydrolysate, 1.45% m/v yeast extract/peptone, 1% v/v ethanol, pH 5.5 after 15 days	5.69	Soares da Silva et al.^17^
*Leifsonia* sp. CBNU-EW3	HS medium supplemented with glucose as carbon source at a final concentration of 20 g/L and yeast extract (5 g/L) as nitrogen source, pH 5 for 15 days in static culture at 30 °C	4	Velmurugan et al.^42^
*Gluconacetobacter xylinus*	3 g/L tea, 20 g/L mannitol and 40 g/L corn steep liquor after 7 days at 30 °C	3.34	Nguyen et al.^35^

### BNC characterization

Following the harvesting and purification of the BNC, the obtained white pellicles were then subjected to quantitative and qualitative analyses.

### Solubility in water and standard organic solvents

The solubility of BNC produced by *Bacillus tequilensis* strain SEE-12 was investigated in water, standard organic solvents (Water, ethanol, chloroform, DMSO, propanol, xylene, methanol, butanol, isopropanol, acetic acid), amonia solution and a mixture of 7% NaOH/ 12% urea/ 81 distilled water^92^. Water, organic solvents and amonia solution fail to dissolve the BNC produced by *Bacillus tequilensis* strain SEE-12. Only, the mixture from alkali hydroxide, urea, and distilled water (7% NaOH/12% urea/81 distilled water) can dissolve cellulose the BNC. The high polarity and strong intermolecular hydrogen bonding within BNC lead to its poor solubility in water and standard organic solvents^93^.

### Scanning electron microscopy (SEM)

**Figure 6:**
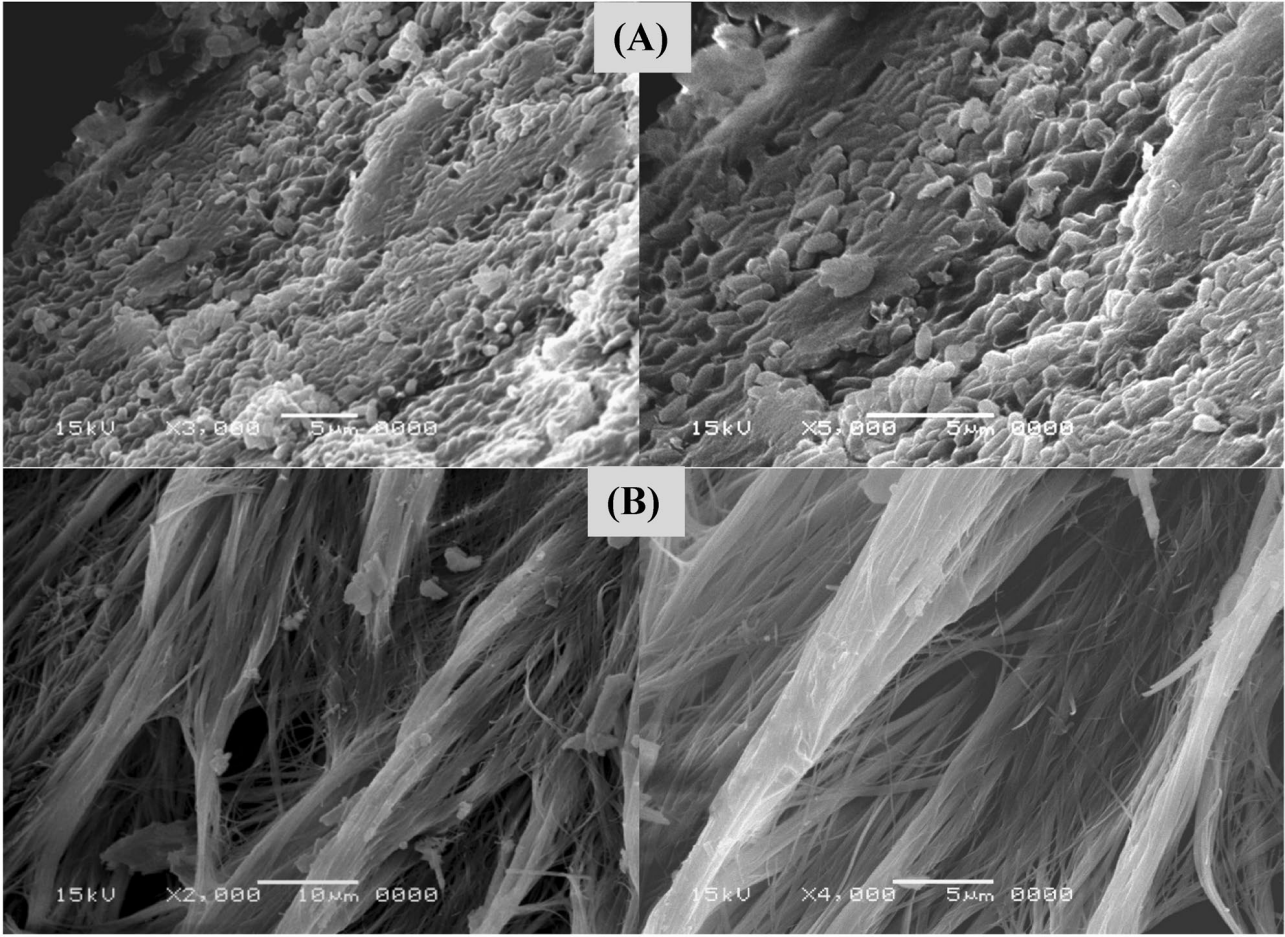
**(A**) SEM electron micrograph for *Bacillus tequilensis* strain SEE-12 trapped in bacterial cellulose network; (**B**) scanning electron microscopy morphology of BNC sample.

The detailed structural and morphological characteristics of bacterial cellulose sample was evaluated using SEM micrographs as shown in Fig. 6. Figure 6A displays the SEM image of *Bacillus tequilensis* strain SEE-12 trapped in the bacterial cellulose. After fermentation, the BNC pellicles which were synthesized were harvested, treated with 0.1 M NaOH solution for 3 h at 80 °C and washed thoroughly with distilled water. The BC morphology has been investigated using both SEM and TEM. The SEM images (Fig. 6B) shows arrangement of nanocellulose. The bacterial nanocellulose exhibited a heterogeneous interconnected network structure composed of randomly oriented nanocellulose^94^.

### Transmission electron microscopy

**Figure 7:**
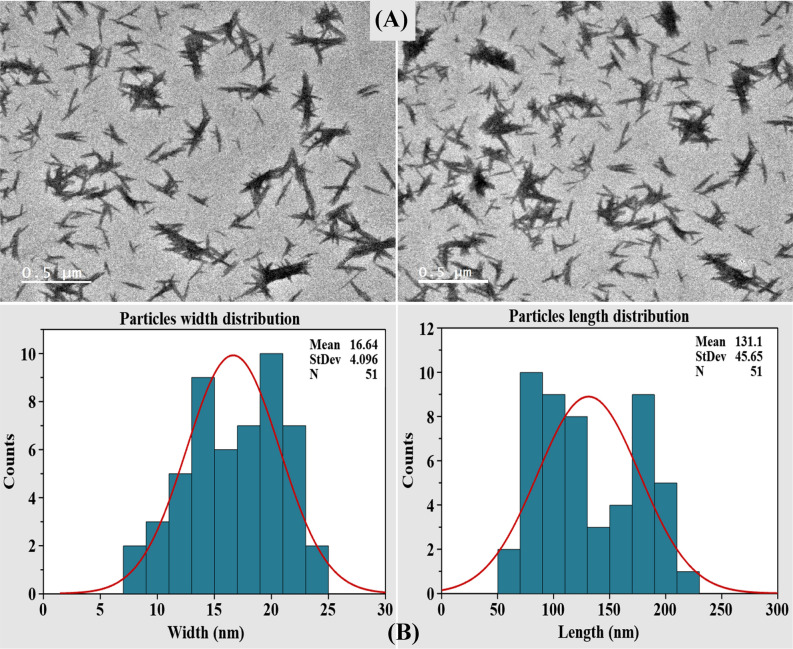
(**A**) Transmission electron microscopy morphology of BNC sample; (**B**) the histograms of length and width distributions for the produced BNC.

Transmission electron microscopy (TEM) has been used as effective tool to reveal the size and shape of BNC produced by *Bacillus tequilensis* strain SEE-12. The TEM images (Fig. 7A) show needle-shaped particles with diameters of 8.54 ‒ 24.91 nm and lengths of 65.36‒217.3 nm. Some nanoparticles were agglomerated into bundles. Generally, agglomeration occurs as a result of Van der Waals forces amongst nanoparticles^97^. ImageJ/Fiji Software, Version 1.48 was used for measuring the size of BNC particles obtained from TEM images. In order to provide an adequate statistical analysis, the length (major axis), and the width (minor axis) were measured. Minitab Statistical Software (Version 19) was used for statistical analysis of the measurement results. The histograms of length and width distributions for BNC particles were plotted and shown in Fig. 7B. The means of the particles width and length were 16.64 and 131.1 nm, respectively.

### Energy dispersive x-ray diffraction (EDX)

Materials' characteristics are always proportional to their molecular structure and composition. The selected micrographs of the surface of bacterial cellulose samples were investigated by use Energy dispersive x-ray diffraction (EDX) attached with FESEM to reveal its elemental composition^38^. In this study, The EDX spectrum (Supplementary Figure S4) shows the nanocellulose possesses carbon and oxygen with percentages of 48.56% and 51.44% respectively.

### Fourier transformed infrared spectroscopy (FTIR)

**Figure 8:**
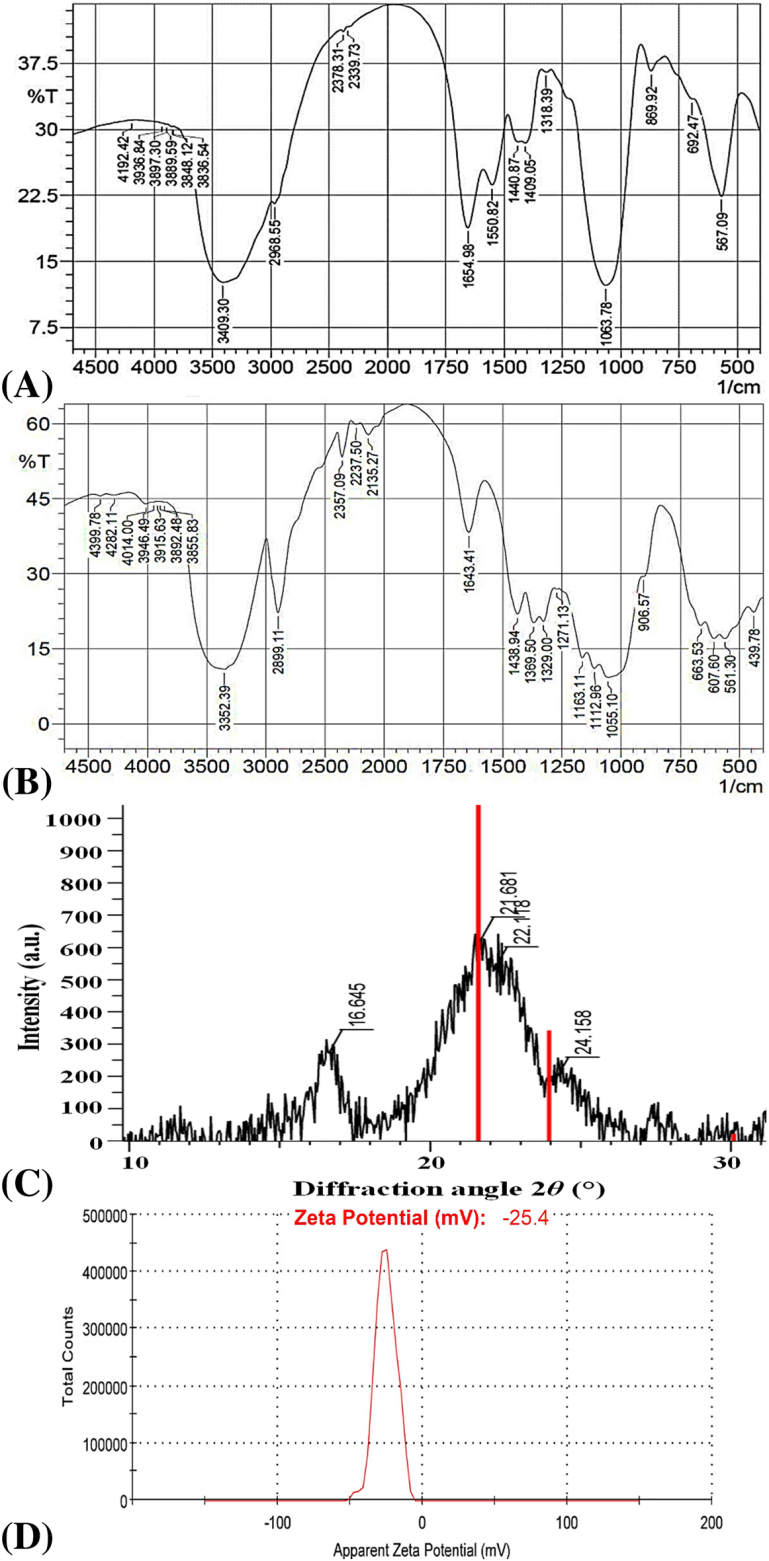
FTIR spectra of BNC sample (**A**) andAvicel PH101(**B**). X-ray diffraction (**C**) and Zeta potential (**D**).

FTIR spectroscopy is a powerful tool for studying the physicochemical properties of polysaccharides. The absorption bands observed in Fig. 8 shows the spectra of BNC (Fig. 8 A); and Avicel PH101 (Fig. 8B) for the analysis of functional groups in their structures. The distinctive bands of the cellulose crystal structure were reported via the Fourier transform infrared spectrum by the presence of sharp and steep band at 1063 cm^−1^ assigned to C–O–C group of carbohydrate skeleton, which shifted to 1055 cm^−1^ for Avicel PH101^98^. In addition, β-glycosidic linkage of cellulose ring assigned at 906 and 869 cm^−1^^99^. On the other hand, bands at 893 ‒1105 cm^−1^ corresponds to the stretching vibration of β-glycosidic linkage at cellulose ring^98^. Bacterial cellulose spectra usually present bands at 1550, 1440 and 1318 cm^−1^ attributed to C − H stretching of CH_2_ and CH_3_ groups^100^. Besides the data described above, the band at 1643 and 1654 cm^−1^ can be attributed to N–H from amide I group of the bacterial cellulose protein. As well as, in pure bacterial cellulose, a broad band at 3,409 cm^−1^ is attributed to O–H stretching vibration, which shifted to 3352 cm^−1^ in Avicel PH101^101^. The main difference between bacterial cellulose and Avicel PH101 samples is an observed increase in the bands around 2900 cm^−1^ assigned to the C–H stretching, due to the presence of the CH and CH_2_ groups^102^ of the cellulose and Avicel PH101. The peak at 2960 cm^−1^ also represents amorphous nature of bacterial cellulose. The band at 3352 cm^−1^ can be attributed to the characteristic band of cellulose (type I) for the stretching vibration of O‒H groups^58^. The peaks around in 3400 to 3500 cm^−1^ is attributed to O–H stretching for pure cellulose^103^.

### X-ray diffraction

The cellulose in fibrous plants is a crystalline unit that is found in their cell walls. One way for determining the crystalline structure of cellulose samples is to employ XRD characterization to identify the crystalline structure of the sample in the form of powder^104^. Figure 8C shows the diffractogram of nanocellulose has three diffraction peaks; 2θ = 16.65°; 21.68°; 22.11°. The peaks are correspond to typical crystalline character of cellulose, which has antiparallel structure^105,106^. The crystallinity index (C_I_) for nanocellulose was (48.64%) that calculated by using Eq. 3 in Supplementary Information. I_am_ is the intensity value for the amorphous cellulose (2θ = 16.65°) , and I_002_ is the intensity value for the crystalline cellulose (2θ = 21.68°). Additionally, the intensity of peaks in FTIR spectra at 1,440 and 1,409 cm^−1^, which correspond to CH_2_ bending, can be used to determine crystallinity index (C_I_) of cellulose sample by quantifying the crystalline fraction of a cellulosic sample in terms of a relative percentage as shown in Eq. 4 in Supplementary Information. Where, I_1440_ and I_1409_ correspond to the FTIR intensity of the indicated bands at 1,440 and 1,409 cm^−1^, respectively. According to the previous equation the crystallinity index (C_I_) for nanocellulose was (50.09%).

### Zeta potential analysis

Net charge is a critical property of nanoparticles that can affect their stability and diffusion rate. Therefore, the electrical characteristics of the BNC particles with high dispersibility were examined by estimating the particles' ζ-potential using electrophoretic mobility measurements. In general, the majority of cellulosic fibres have a negative charge in water. As shown in Fig. 8D, the bacterial nanocellulose possess a negatively charged surface with zeta potential value of − 25.4 mV, indicating a very stable suspension of bacterial nanocellulose in aqueous solvent without particle sedimentation. The zeta potential is used to quantify the repulsive and attractive forces between nanoparticles dispersed in liquid, that provide insight into the stability of the suspension and, indirectly, its capacity for agglomeration. When the zeta potential value is large, the dispersion becomes more stable. While aggregation occurs more frequently when the zeta potential is near 0 mV. In general, nanoparticles in a dispersion with zeta potential values greater than or equal to + 30 mV or − 30 mV exhibit a high degree of stability. Dispersions with a zeta potential value of less than + 25 mV or larger than − 25 mV will eventually aggregate as a result of interparticle interactions which including hydrophobic interactions and Van der Waals forces amongst nanoparticles, as well as hydrogen bonding. The zeta potential is a critical tool for characterizing the surface of a nanoparticle and evaluating its stability in a solution^107^.

### Thermo gravimetric analysis (TGA) and differential scanning calorimetry (DSC)

**Figure 9:**
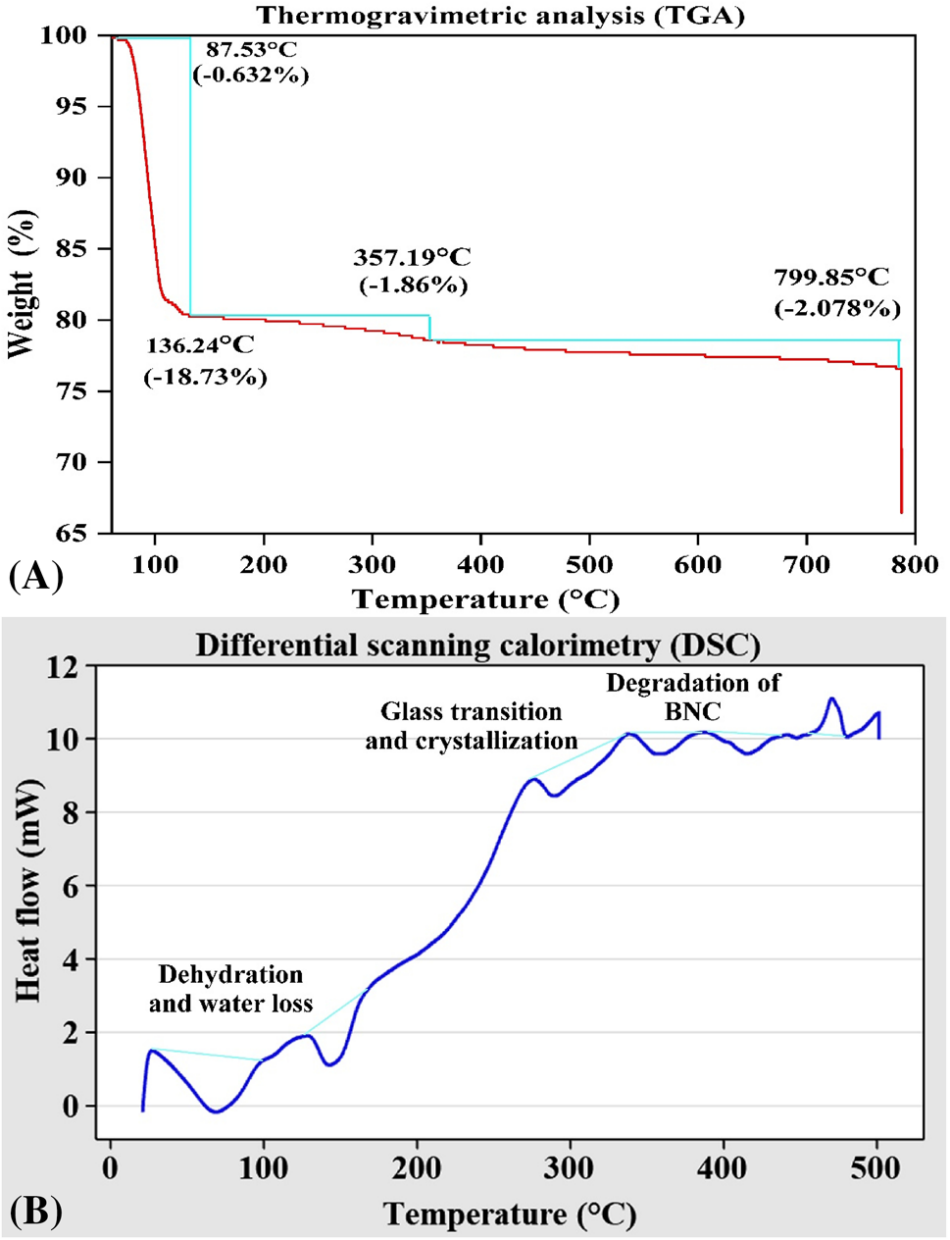
(**A**) Thermogravimetric analysis (TGA), and (**B**) differential scanning calorimetry (DSC) plots of BNC sample.

It is critical to understand the thermostability of nanocellulose, for example, when this material is applied as a filler in a biopolymer. Raw cellulose has intermediate thermal characteristics, with a disintegration temperature of between 315 and 4000 degrees Celsius^40^. As is shown in Fig. 9A, thermogravimetric curves of nanocellulose sample has their own characteristics. The TGA curve started with minor mass losses across ambient temperature to 87.53 °C, which corresponded to the evaporation of partially bound water from the sample. As the temperature is raised higher, two phases of decomposition appeared. A second, substantial weight loss occurred with 88.29–136.24 °C, which may be attributable to the loss of water. While the BNC showed a decomposure rate of 1.862% at 357.19 °C, which could be caused by cellulose degradation including the dehydration, depolymerization and glucose units decomposition. FTIR analysis revealed the presence of hydrogen bonds in the cellulose sample (Fig. 8). In the curve for nanocellulose sample, a significant decomposition was detected, consistent with degradation processes such as dehydration, depolymerization, and glycosyl ring disintegration, followed by a charred residue formation^12^. Mass loss rate was very low above 700 °C (exhibited a decomposition rate of 2.078% was shown at 799.85 °C). These results conferred high thermal stability to the BNC produced by *Bacillus tequilensis* strain SEE-12. Thermal degradation is influenced by structural factors such as crystallinity, fiber arrangement and molecular mass. The thermal stability criterion is a maximum decomposition temperature^40^.

Additionally, the thermal degradation of the cellulose component can be determined using DSC curves, as indicated in Fig. 9B. It can be seen from the DSC curve that the BNC sample contained five peaks. The first transformation peak at 68.18 °C is the thermal effect of dehydration and water loss from the sample. The second peak at 142.3 °C can be attributed to dehydration and water loss or the melting of the crystalline phase of cellulose. The peak at 289.46 °C can be attributed to glass transition (T*g*) and crystallization. The peaks at 357.19 and 415.43 °C can be attributed to degradation of BNC. The maximum peak temperature under the DSC analysis of the BNC at 470.88 °C, and this indicates that the stabilization and structural stability of the BNC compared to other reported BNC. The bacterial cellulose sample was stable up to 200 °C above which decomposition started with a pronounced disintegration at 338.13°C^108^. The thermal stability of bacterial cellulose up to 200 °C could be attributed to its crystallinity and high molecular weight^109^ while the low thermal stability of bacterial cellulose could be as a result of hydrolysis yielding low molecular weight oligosaccharides.

### Particle size analysis

The particle size distributions of bacterial cellulose suspensions were investigated in this study using a particle size analyser (PSA). The size distribution of BNC was determined at a concentration of 0.05% in water as described by Phanthong et al.^110^*.* The experiment was performed at room temperature and included a wavelength range of 10 nm to 4000 nm. The particle size analyzer measurement of a sample of BNC powder dispersed in water revealed a narrow and sharp peak at 365 nm diameter at θ = 11.1° and 476 nm at θ = 90° as shown in Supplementary Figure S5. The sizes recorded by particle size analyser can only be taken as a relative value and cannot be compared with that determined by SEM^111^. SEM capable of detecting geometric dimensions of the particles given by measuring the width of individual particles from the image, and determining their shape and surface structure (e.g. texture)^112,113^. Imaging was favored because of its high-resolution visualization of particles and the minimal effect of artifacts on size determination^113^.

## Supplementary Information

Supplementary Information.

## Data Availability

The 16S rDNA gene sequence has been deposited under the accession number of MN826325 in the Gen Bank database. All data generated or analysed during this study are included in this published article.
