# Supplementary material for: Exploration of a novel and efficient source for production of bacterial nanocellulose, bioprocess optimization and characterization
Source: Sci Rep. 2022 Nov 2;12:18533. doi: 10.1038/s41598-022-22240-x (PMC9630512; doi:10.1038/s41598-022-22240-x)
Supplement: Supplementary file 1 — Supplementary Information. [file 41598_2022_22240_MOESM1_ESM.pdf]

Original Article

Exploration of a novel and efficient source for production of bacterial nanocellulose, bioprocess optimization and characterization

[1]Noura El-Ahmady El-Naggar*, [2]Sahar E. El-Malkey,[3]M. A. Abu-Saied, [2]A.B Abeer Mohammed

[1]Department of Bioprocess Development, Genetic Engineering and Biotechnology Research Institute, City of Scientific Research and Technological Applications (SRTA-City), Alexandria, 21934, Egypt.

[2]Microbial Biotechnology Department, Genetic Engineering and Biotechnology Research Institute, University of Sadat City, Sadat City, Egypt.

[3]Polymeric Materials Research Department, Advanced Technology and New Materials Research Institute, City of Scientific Research and Technological Applications (SRTA-City), New Borg El-Arab City 21934, Alexandria, Egypt.

Corresponding Author's information

Dr. Noura El-Ahmady Ali El-Naggar

Address:

Bioprocess Development Department,

Genetic Engineering and Biotechnology Research Institute,

City of Scientific Research and Technological Applications,

New Borg El- Arab City, 21934, Alexandria, Egypt

Tel: (002)01003738444

Fax: (002)03 4593423

E-mail: nouraalahmady@yahoo.com

Results

Supplementary Table S1. Biochemical tests and identification of *Bacillus tequilensis* strain SEE-12

**Characteristics**	**Properties**
Shape of colony	Irregular
Margin	Entire
Elevation	Flat
Shape of growth in LB	In surface
Shape	Rods
Gram Staining	+
Spore	+, oval
H_2O_2 production	+
**Utilization of**	
Glucose	+
Fructose	+
D-mannitol	+
Sucrose	+
Glycine	+
D-sorbitol	+
D-ribose	–
D-xylose	–
Starch	–
D-galactose	–
D-mannose	–
D-raffinose	–
D-maltose	–
D-trehalose	–
CMC utilization	+
Casein hydrolysis	+
Gelatin hydrolysis	+
Starch hydrolysis	+
H_2O_2 production	+

Supplementary Table S2. Plackett–Burman experimental design for evaluation of independent factors for the production of BNC with coded values along with the experimental BNC.

Std no.	Run no.	A	B	C	D	E	F	G	H	J	K	Dry wt. of BNC (g/L)		Residuals
												Actual	Predicted	
2	1	-1	1	1	-1	1	1	1	-1	-1	-1	3.4	3.38	0.02
7	2	1	-1	-1	-1	1	-1	1	1	-1	1	8.45	8.43	0.02
3	3	1	-1	1	1	-1	1	1	1	-1	-1	10.75	10.77	-0.02
12	4	-1	-1	-1	-1	-1	-1	-1	-1	-1	-1	0.2	0.22	-0.02
1	5	1	1	-1	1	1	1	-1	-1	-1	1	17.35	17.05	0.30
4	6	-1	1	-1	1	1	-1	1	1	1	-1	3.95	3.65	0.31
5	7	-1	-1	1	-1	1	1	-1	1	1	1	11.65	11.35	0.31
6	8	-1	-1	-1	1	-1	1	1	-1	1	1	19.3	19.61	-0.31
10	9	-1	1	1	1	-1	-1	-1	1	-1	1	0.25	0.56	-0.31
9	10	1	1	1	-1	-1	-1	1	-1	1	1	1.6	1.62	-0.02
11	11	1	-1	1	1	1	-1	-1	-1	1	-1	0.8	0.78	0.02
8	12	1	1	-1	-1	-1	1	-1	1	1	-1	11.57	11.88	-0.31
**Variable level**		**g/L**	**g/L**	**%, v/v**	**g/L**	**g/L**		*	**%, v/v**	**ºC**	**Days**			
-1		5	5	50	3	1.5	3.6	50	5	30	4			
1		10	10	100	5	2.67	5	100	10	37	7			

The independent factors are: A (peptone; g/L), B (yeast extract; g/L), C (Cantaloupe juice; %, v/v), D (Na_2HPO_4; g/L), E (citric acid; g/L), F (pH); *G (medium volume; mL/250 mL conical flask), H (inoculum size; %, v/v), j (temperature; ºC), K (incubation time; days).

Supplementary Table S3. Face centered central composite design for evaluation of independent factors with coded values along with the experimental BNC.

Std	Run	Type	Variables			Dry wt. of BNC (g/L)		Residuals
			X_1	X_2	X_3	Actual value	Predicted value	
7	1	Fact	-1	1	1	11.44	11.35	0.09
2	2	Fact	1	-1	-1	12.04	12.06	-0.02
8	3	Fact	1	1	1	9.64	9.76	-0.12
1	4	Fact	-1	-1	-1	6.40	6.21	0.19
15	5	Center	0	0	0	21.48	21.77	-0.29
18	6	Center	0	0	0	22.00	21.77	0.23
11	7	Axial	0	-1	0	18.88	19.12	-0.24
19	8	Center	0	0	0	22.52	21.77	0.75
9	9	Axial	-1	0	0	16.80	16.88	-0.08
6	10	Fact	1	-1	1	14.92	14.75	0.17
13	11	Axial	0	0	-1	16.52	16.76	-0.24
20	12	Center	0	0	0	21.20	21.77	-0.57
16	13	Center	0	0	0	22.80	21.77	1.03
17	14	Center	0	0	0	21.20	21.77	-0.57
14	15	Axial	0	0	1	19.40	19.44	-0.04
4	16	Fact	1	1	-1	11.76	11.59	0.17
12	17	Axial	0	1	0	17.80	17.84	-0.04
10	18	Axial	1	0	0	18.80	19.00	-0.20
3	19	Fact	-1	1	-1	8.56	8.66	-0.10
5	20	Fact	-1	-1	1	13.32	13.42	-0.10

Variable	Variable code	Coded and actual levels		
		-1	0	1
pH	X_1	4	5	6
Cantaloupe juice (%, v/v)	X_2	25	50	75
Incubation time (days)	X_3	4	6	8

Supplementary Table S4. Fit summary for the results of FCCCD of BNC production by *Bacillus tequilensis* strain SEE-12.

**Model Summary Statistics**					
**Source**	**SD**	**R^2**	**Adjusted R^2**	**Predicted R^2**	**PRESS**
Linear	5.33	0.0684	-0.1063	-0.6987	830.08
2FI	5.75	0.1189	-0.2877	-5.6955	3271.91
Quadratic	0.52	0.9945	0.9895	0.9859	6.91
**Lack of Fit Tests**					
**Source**	***SS***	***Df***	***MS***	***F*-value**	***P*-value**
Linear	452.89	11	41.17	87.45	< 0.0001*
2FI	428.20	8	53.52	113.68	< 0.0001*
Quadratic	0.35	5	0.07	0.15	0.9713
**Sequential Model Sum of Squares**					
**Source**	***SS***	***Df***	***MS***	***F*-value**	***P*-value**
Linear vs Mean	33.43	3	11.14	0.39	0.7607
2FI vs Linear	24.69	3	8.23	0.25	0.8609
Quadratic vs 2FI	427.84	3	142.61	526.98	< 0.0001*

* Significant values, *df*: degree of freedom, PRESS: sum of squares of prediction error, two factors interaction: 2FI

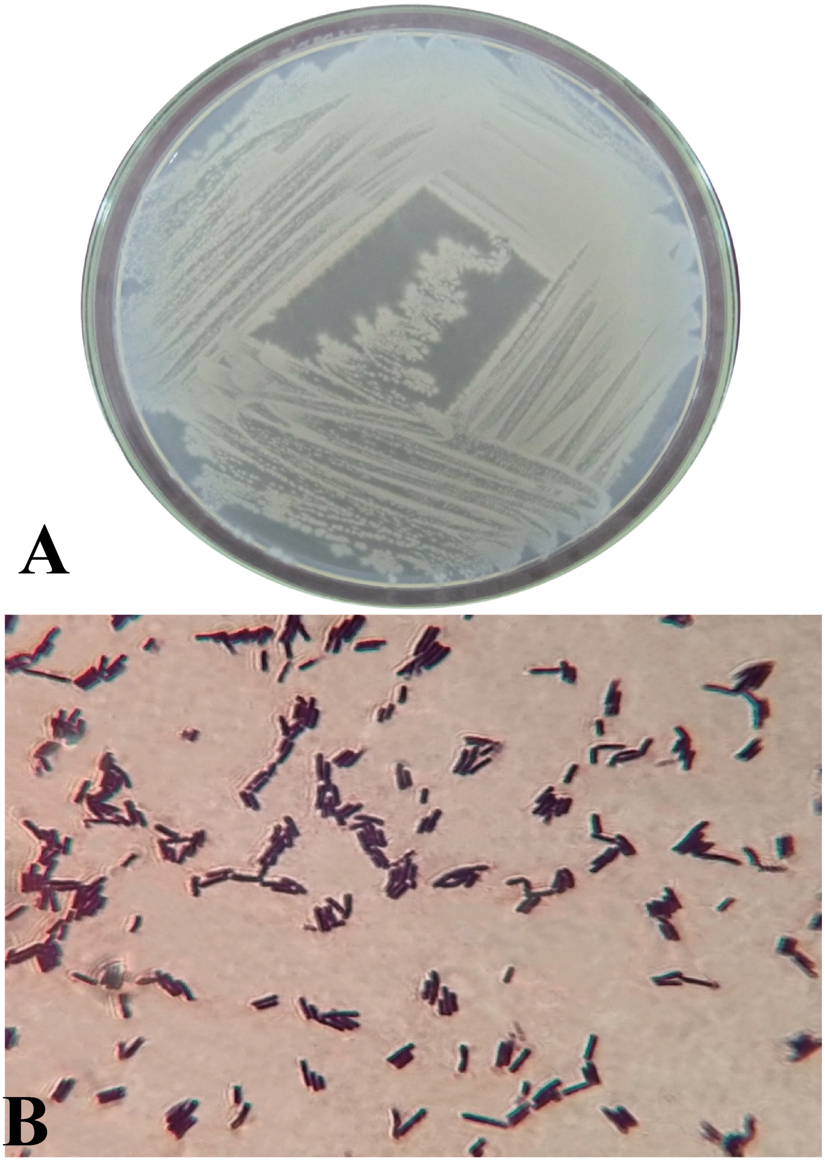

Supplementary Figure S1. A) Cultural characteristics of strain SEE-12 grown on nutrient agar plates; B) Gram stain test shows Gram-positive bacilli.

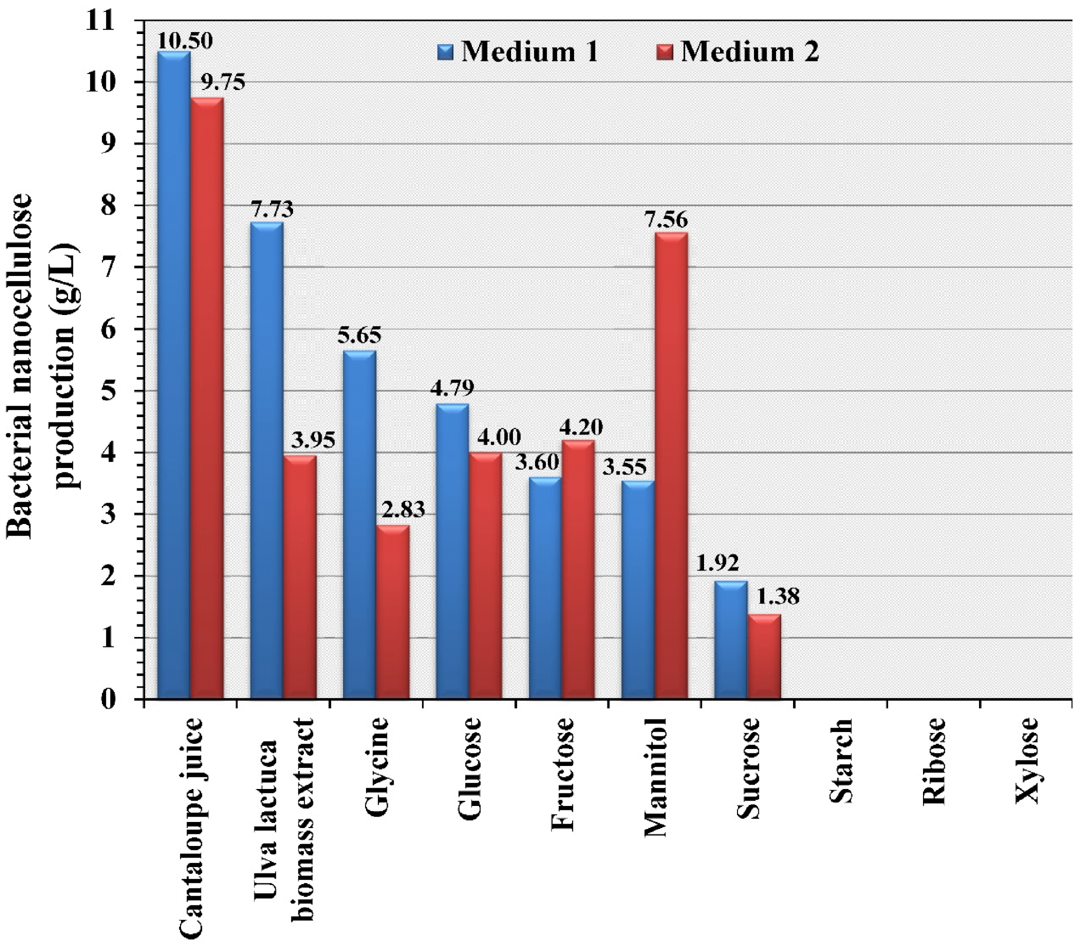

Supplementary Figure S2. Effect of ten carbon sources on bacterial cellulose production.

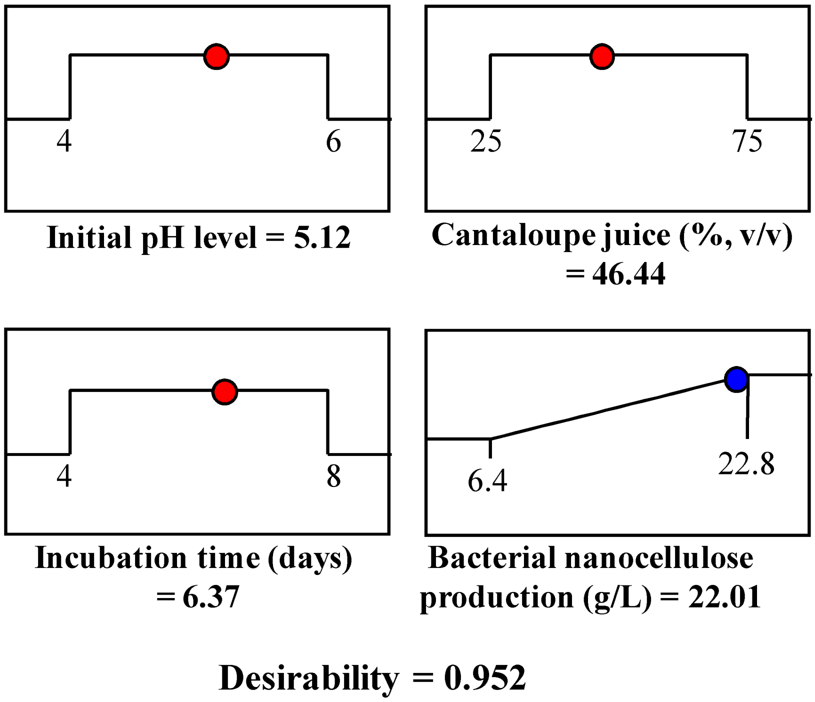

Supplementary Figure S3. The optimization plot displays the optimum predicted values for maximum BNC production and the desirability function.

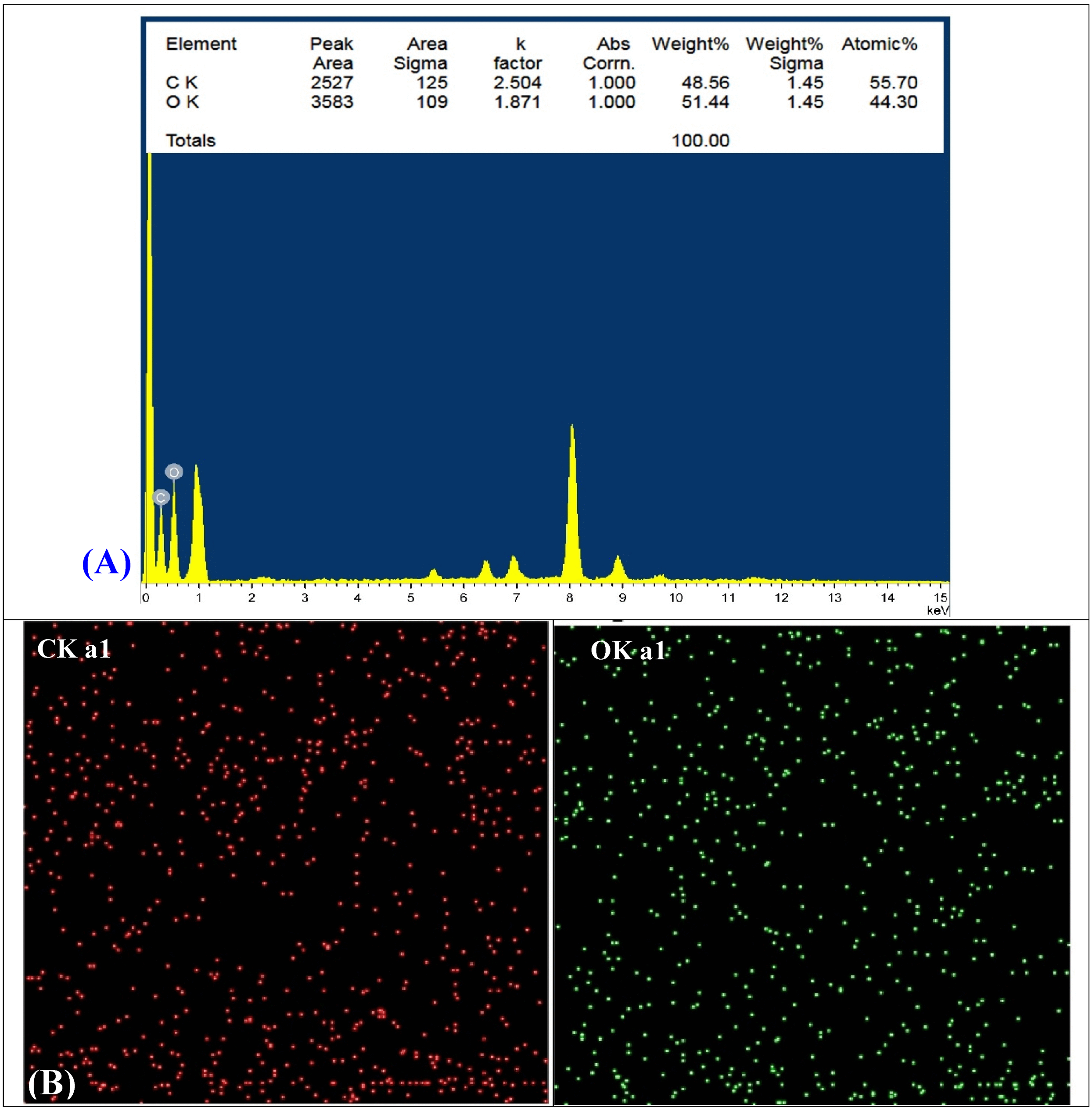

Supplementary Figure S4. EDX analysis and mapping.

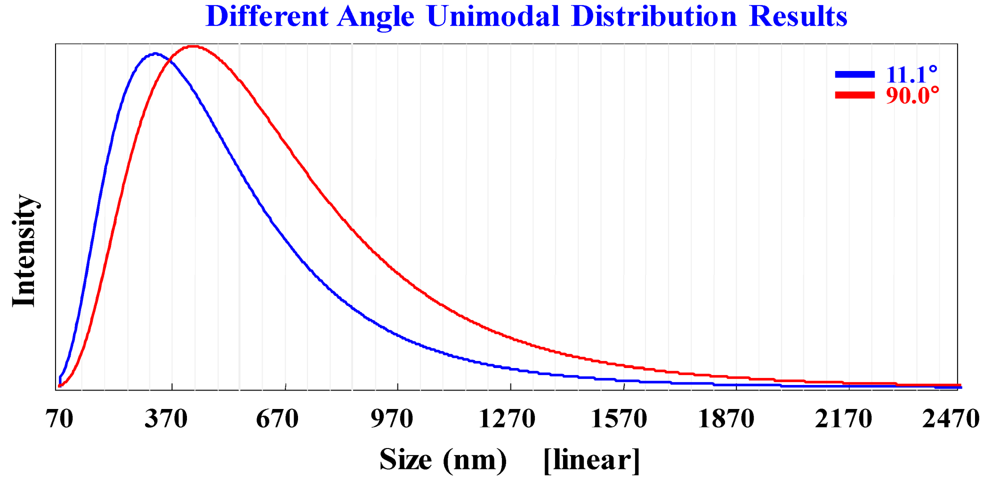

Angle	Mean (nm)	P.I.	Diff. Coef (m^2/s)	Counts/s	Baseline Error	Overflow
**11.1°**	365.1	-20.03	1.17e-12	1.57e+06	0.35%	0
**90.0°**	476.9	-1.77	8.98e-13	2.66e+05	2.85%	0

Supplementary Figure S5. Particle size analysis: Different angle unimodal distribution results

Materials and Methods

Harvesting, purification and quantification of the BNC

After fermentation, the BNC layers which were synthesized and secreted in contact with the air as the exopolysaccharides were harvested after a period of seven to fourteen days of cultivation and treated by the following protocol: washed with water, soaked in a distilled water at 70°C for 3 hours[1], then soaked in 0.1 M NaOH solution for 3 h at 80°C to remove the medium components and dissolve the bacteria cells possibly entrapped in the bacterial cellulose nanofibers. Heating with sodium hydroxide (NaOH) increases viscosity, enhances the removal of specific metabolites, hence promoting purification and cellulose with a low molecular weight is removed, which results in a biomaterial with improved properties[2]. After the BNC turned transparent, the pellicles were washed thoroughly with distilled water to neutralize them (complete alkali removal). The purified BNC was dried at 50°C until it reached a constant weight[3]. The production yield (in g/L dry mass) was determined.

Identification of the bacterial isolate

The most promising bacterial isolate (strain SSE-12) was identified according to its morphological, Gram staining, spore formation, biochemical tests. The bacterial isolate (strain SSE-12) was molecularly identified using 16S rRNA sequencing. Thermo Gene JET Genomic DNA Purification Kit (#K0721) was used to extract. The bacterial genomic DNA. The PCR amplification of the 16S rRNA gene was performed using protocol of El-Naggar *et al.*[4]. The PCR product was purified using the Qiaquick spin-gel extraction kit (Qiagen). The universal primers; 1492R reverse primer (5′-TACGGYTACCTTGTTACGACTT-3′) and 27F forward primer (5'-AGAGTTTGATCCTGGCTCAG-3') were used. The acquired 16S rRNA gene sequence was matched to the publicly available 16S rRNA gene reference sequences in the GenBank databases using the BLASTN[5]. MEGA version X software was used to construct the phylogenetic tree using the neighbour-joining method[6].

Selection of significant variables using Plackett–Burman design (PBD)

PBD[7]is a two-factorial design that defines different process physico-chemical factors necessary to produce high levels of the response with respect to their main effects[8]. In the present work, the variables chosen to be screened by PBD were A (peptone; g/L), B (yeast extract; g/L), C (Cantaloupe juice; %, v/v), D (Na_2HPO_4; g/L), E (citric acid; g/L), F (pH); G (medium volume; mL/250 mL conical flask), H (inoculum size; %, v/v), j (temperature; ºC), K (incubation time;

days) in addition to one dummy variable. Table 2 illustrates the experimental design used to screen the variables. A total of 12 runs of the experiment were performed in order to examine the effects of the chosen factors on the BNC synthesis by *Bacillus tequilensis* strain SEE-12 during static fermentation. The lower and higher levels of the parameters are based on our preliminary research. The experimental design of Plackett–Burman is based on the following first order polynomial equation:

$$Y = \beta_0 + \sum \beta_i X_i$$ **Equation (1)**

Where, Y is the BNC production, β_0 is the intercept for the model and β_i is the linear coefficient, while X_i denotes the independent variable's level.

The Plackett-Burman design does not define the mutual interactions between the process variables; rather, it is employed to screen for and identify significant variables that influence the response[9]. As a result, the face-centered central composite design (FCCCD) was employed to define the levels of significant variables and to investigate the interaction effects among multiple significant variables.

Face centered central composite design (FCCCD)

Face-centered central composite design (FCCCD) is an efficient design that is widely used in optimization processes because it provides a sufficient amount of information for validating accuracy of the model without requiring a large number of experimental runs, thereby lowering the overall cost of the experiment[10]. Based on Plackett–Burman experiment results, FCCCD was used to investigate and optimize the levels and to study the interaction effects among the most significant independent variables that affect the BNC production. These variables namely; pH (X_1), Cantaloupe juice (X_2) and incubation time (X_3). A total of 20 runs were performed in order to optimize the levels and to study the interaction effects among the chosen factors on the BNC synthesis by *Bacillus tequilensis* strain SEE-12. The independent factors were studied at three levels (−1, 0, 1). Table 4 displays the coded levels and actual values of the three variables, as well as the experimentally determined and predicted BNC production results. The center point was repeated six times. The zero levels (central values) chosen for the experimental design were: pH 5, Cantaloupe juice (50 %, v/v) and incubation time (6 days). The twenty runs were carried out in a 250 mL Erlenmeyer flask holding 100 mL of media that had been prepared according to the design. The inoculated media were incubated in an incubator at 37°C. The following second

order polynomial equation was used to fit the experimental data of FCCCD using the regression analysis:

$$Y = \beta_0 + \sum_i \beta_i X_i + \sum_{ii} \beta_{ii} X_i^2 + \sum_{ij} \beta_{ij} X_i X_j \quad \textbf{Equation (2)}$$

Where Y is the predicted BNC production, X_i is the coded levels of independent factors. the β_0, β_i, β_{ii}, β_{ij} denotes the regression, linear, quadratic and β_{ij} the interaction coefficients; respectively.

Statistical analysis

STATISTICA version 8 and Design Expert version 12 for Windows softwares were used for the experimental designs, statistical analysis, and creation of three-dimensional graphs.

SEM and TEM analyses

The size, morphology, and structure of the BNC samples coated by gold sputter coater (SPI-Module) and were examined by a scanning electron microscope (SEM) "JSM-5500 LV; JEOL, Ltd- Japan; by using high vacuum mode operating at 15 kV at the Regional Center of Mycology and Biotechnology, Al-Azhar University, Cairo, Egypt". The samples were also examined with SEM "model JEOL-JSM-IT200; at 20 kV at the Electron Microscope Unit, Faculty of science, Alexandria University, Alexandria, Egypt". The samples were examined with a transmission electron microscope (TEM) "JEM-2100 Plus, JEOL Ltd., Japan; at the Central Laboratory, City of Scientific Research and Technological Applications, Alexandria, Egypt".

Particle size distribution determination

Dynamic laser scattering (DLS) using N5 submicron Beckman Coulter particle size analyzer was used to measure the particle size distribution of the purified BNC. Prior to measurement, the pure BNC sample was sonicated for 15 minutes in an ultrasonic processor.

X-ray Diffraction

X-ray Diffraction (XRD) was employed to evaluate the pattern and crystallinity degree of the BNC. At ambient temperature, the X-ray diffraction patterns were recorded Ni-filtered Cu Kα radiation (λ = 1.54 A°). Diffractometer Type: Bruker D2 Phaser 2nd Gen. The generator current (mA) and operating voltage (kV) were 30 and 10; respectively. Data were collected at a rate of two degrees per minute between 5 and 60 degrees *2*θ. The degree of crystallinity was determined using the empirical method proposed by Segal *et al.*[11] equation from the diffracted intensity data:

$$\mathrm{CI}\ (\%) = \frac{I_{002} - I_{\mathrm{am}}}{I_{002}} \times 100$$

Equation 3

I_{002} is the intensity value for the crystalline cellulose (2θ = 21.68°), and I_{am} is the intensity value for the amorphous cellulose (2θ = 16.65°).

Additionally, the intensity of peaks in FTIR spectra at 1,440 and 1,409 cm^{-1}, which correspond to CH_2 bending, can be used to determine crystallinity index (C_I) of cellulose sample by counting a relative percentage amount of crystalline fraction in a cellulosic sample as shown in Equation (2)

$$\mathrm{C_I}\ (\%) = \frac{\mathrm{I}_{1440}}{\mathrm{I}_{1440} - \mathrm{I}_{1409}} \times 100$$

Equation 4

Where, I_{1440} and I_{1409} represent the FTIR intensities of the particular bands at 1,440 and 1,409 cm^{-1}, respectively.

References

1. Masaoka, S., Ohe, T. & Sakota, N. Production of cellulose from glucose by *Acetobacter xylinum*. *Journal of Fermentation and Bioengineering* **75**, 18–22(1993).
2. Costa, A. F., Almeida, F. C., Vinhas, G. M. & Sarubbo, L. A. Production of bacterial cellulose by *Gluconacetobacter hansenii* using corn steep liquor as nutrient sources. *Frontiers in Microbiology* **8**, 2027(2017).
3. Wu, D. *et al.* Decreased ethyl carbamate generation during Chinese rice wine fermentation by disruption of CAR1 in an industrial yeast strain. *International Journal Food Microbiology* **180**, 19–23 (2014).
4. El-Naggar, N. E., Mohamedin, A., Hamza, S. S. & Sherief, A.-D. Extracellular biofabrication, characterization, and antimicrobial efficacy of silver nanoparticles loaded on cotton fabrics using newly isolated *Streptomyces* sp. SSHH-1E. *Journal of Nanomaterials* Article ID 3257359 (2016).
5. Zhang, Z., Schwartz, S., Wagner, L., & Miller, W. A greedy algorithm for aligning DNA sequences. *Journal of Computational biology* 7(1-2), 203-214 (2000).

6. Kumbhar, J. V., Rajwade, J. M. & Paknikar, K. M. Fruit peels support higher yield and superior quality bacterial cellulose production. *Applied Microbiology and Biotechnology* **99**(16), 6677-6691(2015).
7. Plackett, R. L. & Burman, J. P. The design of optimum multifactorial experiments. *Biometrika* **33**(4), 305-325(1946).
8. El-Naggar, N. E. Extracellular production of the oncolytic enzyme, *L-asparaginase*, by newly isolated *Streptomyces* sp. strain NEAE-95 as potential microbial cell factories: Optimization of culture conditions using response surface methodology. *Curr Pharma Biotechnol* **16**, 162–178 (2015).
9. El-Naggar, N. E., El-Shweihy, N. M. & El-Ewasy, S. M. Identification and statistical optimization of fermentation conditions for a newly isolated extracellular cholesterol oxidase-producing *Streptomyces cavourensis* strain NEAE-42. *BMC Microbiology* **16**, 217 (2016).
10. El-Naggar, N. E. Isolation, screening and identification of *actinobacteria* with uricase activity: Statistical optimization of fermentation conditions for improved production of uricase by *Streptomyces rochei* NEAE–25. *International Journal of Pharmacology* **11**, 644-658 (2015).
11. Segal, L. G. J. M. A., Creely, J. J., Martin, J. r. A. E. & Conrad, C. M. An empirical method for estimating the degree of crystallinity of native cellulose using the X-ray diffractometer. *Textile Research Journal* **29**(10), 786-794(1959).
